# Altered gut microbiome, bile acid composition and metabolome in sarcopenia in liver cirrhosis

**DOI:** 10.1002/jcsm.13342

**Published:** 2023-09-28

**Authors:** Benard Aliwa, Angela Horvath, Julia Traub, Nicole Feldbacher, Hansjörg Habisch, Günter Fauler, Tobias Madl, Vanessa Stadlbauer

**Affiliations:** ^1^ Division of Gastroenterology and Hepatology, Department of Internal Medicine Medical University of Graz Graz Austria; ^2^ Department of Food Science, Nutrition and Technology University of Nairobi Nairobi Kenya; ^3^ Centre for Biomarker Research in Medicine (CBmed) Graz Austria; ^4^ Department of Clinical Medical Nutrition University Hospital Graz Graz Austria; ^5^ Gottfried Schatz Research Center, Molecular Biology and Biochemistry Medical University of Graz Graz Austria; ^6^ Clinical Institute for Medical and Chemical Laboratory Diagnostics Graz Austria; ^7^ BioTechMed‐Graz Graz Austria

**Keywords:** bile acids, cirrhosis, gut microbiome, metabolome, sarcopenia

## Abstract

**Background:**

Sarcopenia in liver cirrhosis is associated with low quality of life and high mortality risk. The pathogenesis has yet to be fully understood. We hypothesized that gut microbiome, bile acid (BA) composition and metabolites differ between cirrhotic patients with and without sarcopenia and contribute to pathogenesis.

**Methods:**

Cirrhotic patients with (*n* = 78) and without (*n* = 38) sarcopenia and non‐cirrhotic controls with (*n* = 39) and without (*n* = 20) sarcopenia were included in this study. Faecal microbiome composition was studied by 16S rDNA sequencing, serum and faecal BA composition by ultra‐high‐performance liquid chromatography–tandem mass spectrometry, and metabolite composition in serum, faeces and urine by nuclear magnetic resonance.

**Results:**

*Bacteroides fragilis* , *Blautia marseille*, *Sutterella* spp. and *Veillonella parvula* were associated with cirrhotic patients with sarcopenia, whereas *Bacteroides ovatus* was more abundant in cirrhotic patients without sarcopenia. We observed significantly elevated secondary BAs, deoxycholic acid (DCA; *P* = 0.01) and lithocholic acid (LCA; *P* = 0.02), and the ratios of deoxycholic acid to cholic acid (DCA:CA; *P* = 0.04), lithocholic acid to chenodeoxycholic acid (LCA:CDCA; *P* = 0.03) and 12 alpha‐hydroxylated to non‐12 alpha‐hydroxylated BAs (12‐α‐OH:non‐12‐α‐OH BAs; *P* = 0.04) in serum of cirrhotic patients with sarcopenia compared with cirrhotic patients without sarcopenia, indicating an enhanced transformation of primary to secondary BAs by the gut microbiome. CA (*P* = 0.02) and the ratios of CA:CDCA (*P* = 0.03) and total ursodeoxycholic acid to total secondary BAs (T‐UDCA:total‐sec‐BAs, *P* = 0.03) were significantly reduced in the stool of cirrhotic patients with sarcopenia compared with cirrhotic patients without sarcopenia. Also, valine and acetate were significantly reduced in the serum of cirrhotic patients with sarcopenia compared with cirrhotic patients without sarcopenia (*P* = 0.01 and *P* = 0.03, respectively). Multivariate logistic regression further confirmed the association of *B. ovatus* (*P* = 0.01, odds ratio [OR]: 12.8, 95% confidence interval [CI]: 168.1; 2.2), the ratios of 12‐α‐OH:non‐12‐α‐OH BAs (*P* = 0.03, OR: 2.54, 95% CI: 0.99; 6.55) and T‐UDCA:total‐sec‐BAs (*P* = 0.04, OR: 0.25, 95% CI: 0.06; 0.98) in serum and stool CA:CDCA (*P* = 0.04, OR: 0.79, 95% CI: 0.62; 0.99), and serum valine (*P* = 0.04, OR: 1.00, 95% CI: 1.02; 1.00) with sarcopenia in cirrhosis after correcting for the severity of liver disease and sex.

**Conclusions:**

Our study suggests a potential functional gut microbiome–host interaction linking sarcopenia with the altered gut microbiomes, BA profiles and amino acids pointing towards a potential mechanistic interplay in understanding sarcopenia pathogenesis.

## Introduction

Sarcopenia is the progressive loss of skeletal muscle mass and function and occurs in more than 40–70% of liver cirrhosis.^1^, ^2^ Sarcopenia can occur with older age (primary) or secondary in the context of inflammatory metabolic diseases.^3^ Sarcopenia is associated with more frequent complications, higher mortality and reduced quality of life in patients with cirrhosis.^2^ However, the pathogenesis of sarcopenia in cirrhosis is not fully understood yet.

Inflammation may be linked to the gut microbiome because the gut microbiome composition of liver cirrhotic patients is altered compared with healthy controls with a decreased diversity and an increase in potential pathogenic species.^4^ This alteration in the gut microbiome composition is associated with increased bacterial translocation and inflammation.^5^ Besides inflammation, hormones regulate muscle atrophy.^3^ Hormones including insulin‐like growth factor 1 (IGF‐1) potentially mediate skeletal muscle protein synthesis and prevent muscle protein degradation.^6^ In addition to responding to changes in hormonal status, skeletal muscle adapts to changes mediated by external stimuli such as nutrition, stress and exercise by secreting myokines including myostatin, fibroblast growth factor 21 (FGF‐21) and irisin.^6^, ^7^

In addition to causing inflammation, the gut microbiome metabolizes and produces important bioactive metabolites including bile acids (BAs) and amino acids.^8^, ^9^ BAs facilitate the uptake of dietary fatty acids and are signalling molecules with diverse endocrine functions through farnesoid X receptor (FXR) and G‐protein coupled receptor.^8^ Altered gut microbiome composition with altered BA profiles is a feature of liver cirrhosis.^10^ Furthermore, liver cirrhosis is associated with high energy demand and amino acid depletion.^11^ Because of amino acid depletion, especially of branched‐chain amino acids (BCAAs), skeletal muscle protein degradation is significantly increased in cirrhotic patients, leading to loss of muscle mass.^7^ BAs and amino acids have been shown to mediate the production of hormones including IGF‐1 and FGF‐21.^12^

The molecular mechanisms mediating sarcopenia in cirrhosis have not yet been fully understood. Most of the research has been conducted in animal models, while clinical studies of sarcopenia in cirrhotic patients are rare. We, therefore, set out to investigate whether sarcopenia in cirrhosis is associated with changes in the gut microbiome composition, BA profile and metabolite composition. In this prospective system‐biology cohort study, we aimed to establish this association to derive novel insights into the pathophysiology of sarcopenia.

## Methods

### Study population recruitment and the study protocol

Consecutive liver cirrhotic patients and non‐cirrhotic controls seen at the Department of Gastroenterology and Hepatology of the Medical University of Graz between April 2017 and January 2019 were assessed for eligibility using convenience sampling. Male and female patients over the age of 18 years, who gave written informed consent, with histological or clinical/radiological diagnosis of cirrhosis and a computed tomography/magnetic resonance imaging (CT/MRI) scan within ±2 months of the baseline study visit, were included into the cirrhosis cohort. Exclusion criteria included hepatic encephalopathy > grade 2 and/or other cognitive disorders not allowing for informed consent, hepatocellular carcinoma stage Barcelona Clinic Liver Cancer stage C or D (BCLC C or D), ursodeoxycholic acid (UDCA) treatment and current intake of probiotics and antibiotics. The control group included non‐cirrhotic patients diagnosed with diseases listed in the footnote (*Table* *1*), who were seen at the same time at the Department of Gastroenterology and Hepatology of the Medical University of Graz. Male and female patients over the age of 18 years, who gave written informed consent and who had a CT/MRI scan within ±2 months of the baseline study visit, were included into the control group. Exclusion criteria included the diagnosis of cirrhosis, disorders not allowing for informed consent and current intake of probiotics and antibiotics. Sarcopenia was diagnosed according to the European Working Group of Sarcopenia in Older People (EWGSOP) 2010 criteria that recommend measurement of loss of muscle mass, muscle strength and function as the diagnostic criteria.^13^ The loss of muscle mass was assessed based on the cross‐sectional CT/MRI imaging of the L3 vertebra. Low muscle mass was defined as L3‐muscle area of ≤52.4 cm^2^/m^2^ in males and ≤38.5 cm^2^/m^2^ in females.^13^ Muscle strength was determined by measuring handgrip strength using a hydraulic dynamometer (Jamar Hydraulic Hand Dynamometer), and the cut‐off values were <27 kg for men and <16 kg for women.^13^ Muscle function was determined by measuring gait speed. For this test, the time used to pass a 4‐m course marked on the hallway was recorded in seconds, and the cut‐off value of speed ≤ 0.8 m/s was considered.^13^

**Table 1:** Characteristics of cirrhotic patients with and without sarcopenia

	Cirrhosis with sarcopenia (*n* = 78)	Cirrhosis without sarcopenia (*n* = 38)	*P*‐values
Gender (*n*/%)	Male, 64 (82.1%); female, 14 (17.9%)	Male, 22 (57.9%); female, 16 (42.1%)	0.007
Age (years)	64 (61; 68)	62 (60; 67)	NS
Body mass index (kg/m^2^)	25.7 (24.2; 27.1)	29.5 (27.4; 31.2)	<0.0001
Charlson comorbidity index	5 (5; 6)	5 (4; 6)	NS
Muscle mass and function			
Muscle mass (cm^2^/m^2^)	39.80 (37.58; 41.17)	55.58 (49.70; 58.63)	<0.0001
Handgrip strength (kg)	29.15 (27.00; 31.00)	30.33 (26.33; 33.60)	NS
Mid‐arm muscle circumference (cm)	23.6 (23.1; 24.6)	27.4 (25.7; 28.1)	<0.0001
Triceps skinfold thickness (mm)	12.15 (11.00; 13.60)	12.10 (10.20; 14.40)	NS
Gait speed (m/s)	0.92 (0.84; 1.00)	1.02 (0.91; 1.07)	NS
Chair rise (s)	17.95 (16.41; 19.60)	14.68 (14.02; 19.28)	NS
Liver functions			
MELD score	11 (10; 14)	11 (9; 12)	NS
Bilirubin (mg/dL)	0.70 (0.55; 0.96)	0.38 (0.30; 0.57)	NS
Albumin (g/dL)	3.5 (3.4; 3.7)	3.6 (3.4; 3.9)	NS
Total protein (g/dL)	7.2 (7.7; 7.5)	7.2 (6.9; 7.4)	NS
Prothrombin time (INR)	1.23 (1.16; 1.32)	1.21 (1.15; 1.28)	NS
AST (U/L)	46 (43; 52)	52 (47; 89)	NS
ALT (U/L)	28 (26; 35)	40 (34; 59)	0.041
GGT(U/L)	131 (96; 156)	113 (92; 164)	NS
Other routine laboratory parameters			
CRP (mg/dL)	5.5 (3.4; 9.5)	6.1 (2.8; 7.8)	NS
Haematocrit (%)	33.2 (31.2; 35.6)	36.5 (34.4; 39.4)	0.044
Creatinine (mg/dL)	0.89 (0.81; 99)	0.91 (0.84; 1.02)	NS
Urea (mg/dL)	40.0 (35.0; 47.0)	32.0 (30.0; 44.0)	NS
Gut permeability, bacterial translocation and inflammation			
Zonulin (ng/mL)	98.1 (80.8; 110.3)	113.4 (92.4; 162.0)	NS
DAO (U/mL)	19.50 (16.23; 25.40)	17.37 (14.09; 20.77)	NS
Calprotectin (μg/g)	101.1 (75.4; 126.7)	58.4 (40.8; 116.0)	NS
LBP (μg/mL)	19.21 (17.71; 22.30)	17.73 (16.74; 22.02)	NS
sCD14 (μg/mL)	1.75 (1.67; 1.87)	1.76 (1.70; 1.84)	NS
Muscle biomarkers			
Myostatin (ng/mL)	36.45 (34.01; 40.47)	43.27 (36.08; 47.36)	NS
Irisin (μg/mL)	1.48 (1.33; 1.88)	1.81 (1.41; 2.13)	NS
FGF‐21 (ng/mL)	0.36 (0.25; 0.48)	0.27 (0.15; 0.37)	NS
IGF‐1 (ng/mL)	55.89 (44.37; 65.70)	51.74 (43.36; 76.35)	NS

*Note*: The data shown are absolute numbers and percentages, or median and 95% confidence interval (lower; upper). *P* < 0.05 values were obtained from the Student *t*‐test or Mann–Whitney *U* test. The control group included non‐cirrhotic patients diagnosed with chronic kidney disease, osteoporosis, pancreatitis, hypertension, hypercholesteraemia, diabetes type II, Hashimoto thyroiditis, chronic obstructive pulmonary disease, thrombocytopenia, ascites, portal hypertension, splenomegaly, reflux esophagitis and appendicitis. Abbreviations: ALT, alanine aminotransferase; AST, aspartate aminotransferase; CRP, C‐reactive protein; DAO, diamino‐oxidase; FGF‐21, fibroblast growth factor 21; GGT, gamma‐glutamyl transferase; IGF‐1, insulin‐like growth factor 1; INR, international normalized ratios; LBP, lipopolysaccharide‐binding protein; MELD, model of end‐stage liver disease; NS, not significant; sCD14, soluble CD14.

Gut permeability and bacterial translocation biomarkers (zonulin and calprotectin in stool, and diamino‐oxidase [DAO], lipopolysaccharide [LPS], lipopolysaccharide‐binding protein [LBP] and soluble CD14 [sCD14] in serum) and muscle‐related biomarkers (IGF‐1, FGF‐1, irisin and myostatin in serum) were quantified using enzyme‐linked immunosorbent assay (ELISA). Additionally, faecal and serum BAs were analysed using ultra‐high‐performance liquid chromatography–tandem mass spectrometry (UPLC‐MS/MS), while metabolite composition in serum, stool and urine was analysed by nuclear magnetic resonance (NMR) spectroscopy, as previously described.^14^ For detailed analytical protocols, see the supporting information.

The gut microbiome composition was studied by faecal 16S rDNA sequencing. DNA was isolated using a MagNA Pure LC DNA isolation kit (Roche, Mannheim, Germany) as per the manufacturer's protocol. V1–2 was amplified (primer sets: forward‐AGAGTTTGATCCTGGCTCAG and reverse‐TGCTGCCTCCCGTAGGAGT; supporting information) and sequenced using Illumina MiSeq technology (Illumina, Eindhoven, Netherlands) as described before.^15^ Batch effects were reduced by randomly assigning the samples to isolation and sequencing batches. Furthermore, negative controls for DNA isolation, library prep and sequencing were run to assess and control for contamination from the reagents and cross‐contaminations during the workflow. The pre‐processing of sequence reads was implemented on QIIME 2 on a local Galaxy instance (https://galaxy.medunigraz.at). Denoising (primers removing, quality filtering, correcting errors in marginal sequences, removing chimeric sequences, removing singletons, joining paired‐end reads and dereplication) was done with DADA2. Reads that exceeded the maximum expected error rate of 2 or contained bases with a quality score below 2 at any instance were excluded from further processing. Taxonomy was assigned based on the Silva V132 database release at 99% operational taxonomic unit (OTU) level with a Naïve Bayes classifier. After pre‐processing, an average of 17.105 (range from 3.525 to 34.816) sequence reads per sample were obtained. The OTU table in biom format and metadata of the study participants were uploaded into Calypso 8.84 (http://cgenome.net/wiki/index.php/Calypso) for further processing and analysis.^16^ The OTU table was filtered and normalized to remove Cyanobacteria and Chloroplasts, samples with <1000 sequence reads and taxa with <0.01 relative abundance across all samples. The gut microbiome community richness was determined by Chao1 and principal coordinates analysis (PCoA) as a measure of alpha and beta diversity, respectively. Linear discriminant analysis (LDA) effect size (LEfSe) and analysis of the composition of the microbiome (ANCOM) were used to identify OTUs associated with sarcopenia in cirrhosis and to assess the significance of the differences in the OTU abundances between the groups, respectively. The sequencing data are available at the NCBI Sequencing Read Archive (accession number, PRJNA933898, https://www.ncbi.nlm.nih.gov/sra/PRJNA933898). For functional predictions, the OTU abundance table with valid taxonomy identifiers and metadata were uploaded into MicrobiomeAnalyst 2.0 (accessible at https://www.microbiomeanalyst.ca/) for further processing, which involved filtering, rarefaction normalization and sample inferencing as described in [17]. The taxonomic assignments in this analysis were made using the SILVA reference database. Tax4Fun 2 was employed to identify the gut microbiome‐predicted functional profiles associated with cirrhotic patients with and without sarcopenia and non‐cirrhotic controls with and without sarcopenia.

We investigated selected BA ratios reflective of the gut microbiome and liver enzymatic activities. The ratio of cholic acid to chenodeoxycholic acid (CA:CDCA) was selected as an indicator of a potential shift in BA synthesis from the classical pathway to the alternative pathway.^18^ The ratios of secondary to primary BAs, deoxycholic acid to cholic acid and lithocholic acid to chenodeoxycholic acid (DCA:CA and LCA:CDCA, respectively), were analysed as an indicator of BAs microbial transformation associated with altered secondary BAs production.^18^ To judge the theoretical potential expression of the gut microbiome‐derived enzymes critical for BAs microbial transformation, we measured BAs gene abundances. BA primers were synthesized and supplied by Eurofins Genomics (Vienna, Austria; *Table* *S2*). The study was approved by the research ethics committee of the Medical University of Graz (29‐280 ex 16/17), registered at clinicaltrials.gov (NCT03080129) and conducted according to the Declaration of Helsinki (revised version 2013).

### Statistical analysis

Descriptive and comparative statistics were used to assess and compare data between cirrhotic patients with sarcopenia and cirrhotic patients without sarcopenia, as well as controls with and without sarcopenia. For categorical variables, the *χ* ^2^ test was used to determine the significant differences between the two groups. Furthermore, for continuous data, the Student *t*‐test was used to evaluate the significance of the differences between the two groups. Where assumptions of parametric testing were not met, the Mann–Whitney *U* test was used as an alternative to the Student *t*‐test. We conducted multiple hypothesis tests to explore various research questions and assess the relationships between different variables. To avoid false positive results due to multiple testing, *P*‐values were corrected for multiple testing according to the Benjamini–Hochberg method. By setting a stringent alpha level of only 5%, we aim to reduce the chances of reporting false positives.

To mitigate the influence of potential confounders, we employed several strategies. We identified and corrected for drug use (proton pump inhibitor, beta‐blocker and diuretics), severity of disease and sex as the most relevant confounders for our analysis.^5^, ^19^ Furthermore, we recruited a non‐cirrhotic control group based on potential confounding variables.

Regularized logistic least absolute shrinkage and selection operator (LASSO) regression was used to preselect features associated with sarcopenia in cirrhosis for high‐dimensional and correlated data. The dataset was randomly partitioned into two sets (70% training and 30% test). The training dataset was used to train the model and predictors selection, thereafter, using the predictors selected by the training dataset the model was fit to the test dataset. Cross‐validation was used to select the optimal lambda value (regularization parameter) and to prevent overfitting and underfitting. Furthermore, the K‐fold cross‐validation technique was conducted to prevent random changes in the optimal lambda value selected during the cross‐validation. Similarly, features were combined to make them more informative and reduce the risk of overfitting. Outliers were removed to prevent undue influence on the LASSO model's performance. The LASSO regression model was cross‐validated and hyper‐tuned using the best obtained lambda. Ten‐fold cross‐validation was conducted to avoid random changes in the lambda value selected during the cross‐validation. The LASSO estimated coefficients were used to determine the influence of covariates in sarcopenia prediction. Additionally, multivariate logistic regression was used to build models that independently predicted sarcopenia even after adjusting for possible confounders.

The MetaboAnalyst 5.0 (https://www.metaboanalyst.ca) was employed for conducting metabolite set enrichment analysis (MSEA) and pathway analysis, enabling the identification of significantly different metabolite sets and pathways that exhibit biologically relevant patterns between compared groups. The serum, urine and stool metabolomics data containing metabolite abundances were uploaded onto the MetaboAnalyst. The data were normalized to ensure quality and comparability. Metabolites were identified based on chemical names and annotated based on the Human Metabolome Database (HMDB) and Kyoto Encyclopedia of Genes and Genomes (KEGG) metabolite set library. Spearman's method performed the correlation of differentially significant clinical parameters, BAs, metabolomes and OTUs. Analysis was performed in SPSS Version 25 (IBM Corporation, Armonk, NY, USA), R software 4.0.2 (http://www.R‐project.org) and MetaboAnalyst 5.0 (https://www.metaboanalyst.ca).^20^

## Results

### Clinical and demographic characteristics of the study participants

A total of 175 participants were included in this study: 116 liver cirrhotic patients with (*n* = 78) and without (*n* = 38) sarcopenia and 59 controls with (*n* = 39) and without (*n* = 20) sarcopenia. The baseline clinical and demographic data for all groups are shown in *Tables* *1* and *S3* and *Figure* *S1*. In cirrhosis, significantly more males (82.1%) compared with females (17.9%) were sarcopenic (*P* = 0.007), and therefore, the relative risk (RR) of males developing sarcopenia was 1.5 times higher than for females (*P* = 0.005). In cirrhotic patients with sarcopenia, we observed significantly reduced body mass index (BMI), muscle mass, mid‐arm muscle circumference (MAMC), haematocrit and alanine aminotransferase (ALT) compared with cirrhotic patients without sarcopenia (*P* = 0.0001, *P* = 0.0001, *P* = 0.0001, *P* = 0.044 and *P* = 0.041, respectively). Additionally, among the control group, controls with sarcopenia showed significant reduction in BMI, muscle mass, MAMC, and albumin compared to controls without sarcopenia (*P* = 0.001, *P* = 0.001 and *P* = 0.01, respectively). However, haematocrit and ALT were comparable between controls with and without sarcopenia (*P* > 0.05).

When analysing male and female patients separately, BMI, muscle mass and MAMC were significantly lower in cirrhotic patients with sarcopenia compared with patients without sarcopenia, whereas haematocrit was only different in male patients (*Tables* *S4* and *S5*). However, ALT and albumin in serum were comparable between cirrhotic patients with and without sarcopenia in both sexes (*Tables* *S4* and *S5*). Controls with sarcopenia had significantly reduced BMI, muscle mass and MAMC compared with those without sarcopenia, whereas ALT and haematocrit remained comparable between non‐cirrhotic controls with and without sarcopenia in both sexes. Multivariate regression analysis showed that BMI, muscle mass and MAMC were independently associated with sarcopenia even after adjusting for sex and severity of liver disease (*Table* *4*). Furthermore, these results indicate that BMI, muscle mass and MAMC are associated with sarcopenia irrespective of cirrhosis whereas haematocrit and ALT are cirrhotic‐associated features for sarcopenia, mainly in male patients, possibly due to the lower number of female patients in our study. Contrary to our expectations, stool zonulin, stool calprotectin and serum LBP, sCD14, IGF‐1, myostatin, FGF‐21, irisin and DAO were comparable in cirrhotic patients with and without sarcopenia and in controls with and without sarcopenia (*P* > 0.05). No sex‐specific differences could be observed in gut permeability, bacterial translocation or muscle biomarkers.

### Altered gut microbiome composition in sarcopenia in cirrhosis

**Figure 1:**
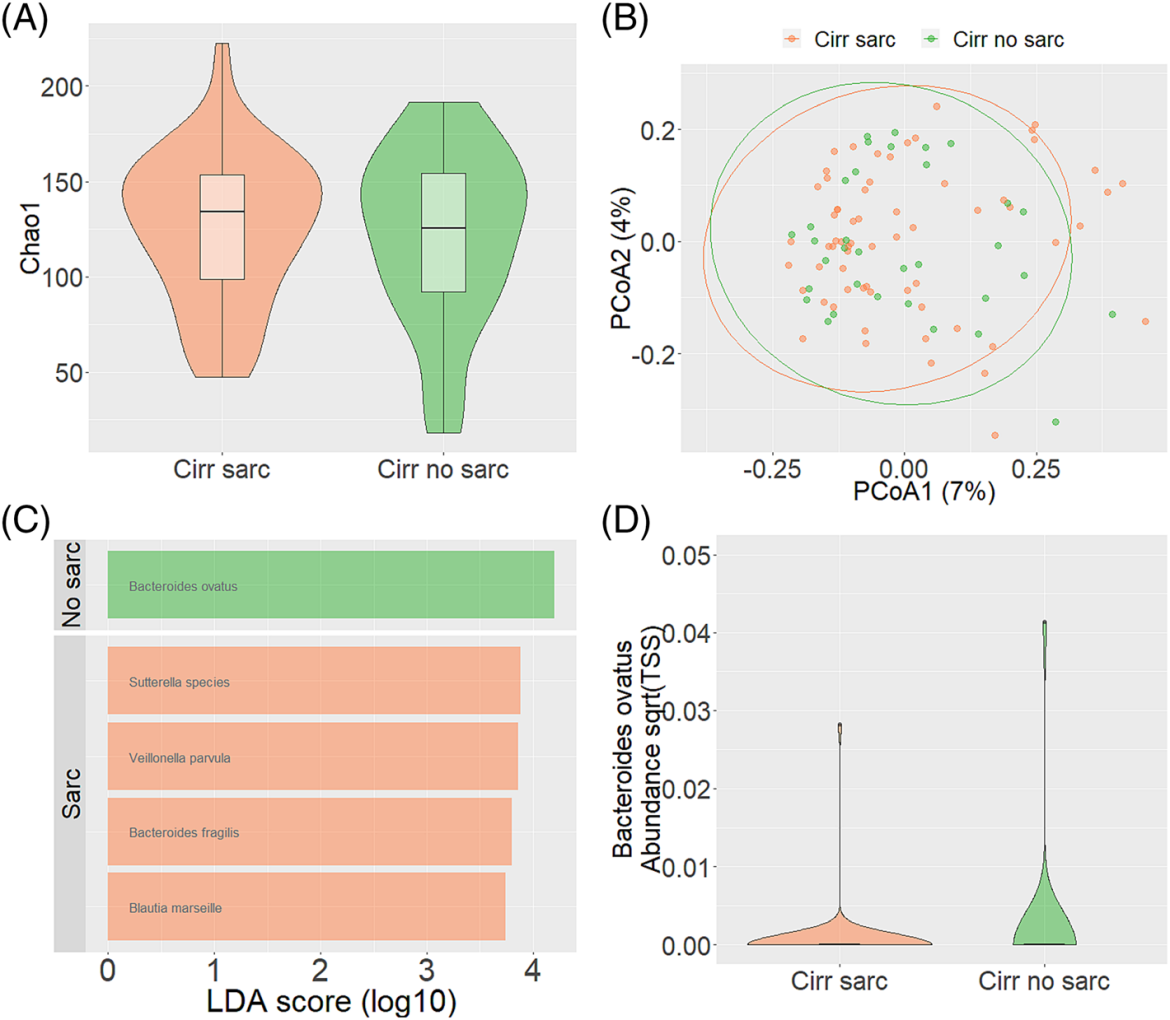
Chao1 index as a measure of alpha diversity between cirrhotic patients with and without sarcopenia (A). Bray–Curtis as a measure of beta diversity between cirrhotic patients with and without sarcopenia (B). Bacteria identified by regularized logistic least absolute shrinkage and selection operator (linear discriminant analysis [LDA] effect size) to be associated with cirrhotic patients with and without sarcopenia (C). Altered *Bacteroides ovatus* abundance between cirrhotic patients with and without sarcopenia (D). PCoA, principal coordinates analysis.

Alpha diversity (Chao1 index: *P* > 0.05; *Figure* *1* *A*) and beta diversity (PCoA; *Figure* *1* *B*) showed no significant difference between cirrhotic patients with and without sarcopenia. However, LEfSe showed that *Bacteroides fragilis* , *Blautia marseille*, *Sutterella* spp. and *Veillonella parvula* were associated with cirrhotic patients with sarcopenia. Conversely, *Bacteroides ovatus* was associated with cirrhotic patients without sarcopenia (*Figure* *1* *C*); ANCOM confirmed *B. ovatus* to be significantly more abundant in cirrhotic patients without sarcopenia compared with cirrhotic patients with sarcopenia (*Figure* *1* *D*). Multivariate logistic regression further confirmed *B. ovatus* to be independently associated with sarcopenia in cirrhosis even when adjusted for the severity of liver disease and drug (proton pump inhibitor, beta‐blocker and diuretics) use (*P* = 0.01; odds ratio [OR]: 12.8, 95% confidence interval [CI]: 168.1; 2.2; *Table* *4*). The relative abundances of *B. ovatus* , *B. fragilis* , *B. marseille*, *Sutterella* spp. and *V. parvula* were comparable between controls with and without sarcopenia, indicating that the alteration of these bacteria is a specific feature for sarcopenia in cirrhosis.

Alpha and beta diversity showed no sex‐specific differences. LEfSe showed that *B. fragilis* was associated with male cirrhotic patients with sarcopenia while *B. ovatus* was associated with male cirrhotic patients without sarcopenia. ANCOM further demonstrated that *B. ovatus* was more abundant in male cirrhotic patients without sarcopenia than those with sarcopenia. Moreover, *V. parvula* was associated with female cirrhotic patients with sarcopenia. Multivariate regression analysis further demonstrated that *B. ovatus* was independently associated with sarcopenia in male cirrhotic patients, even after adjusting for drug use. These results indicate that sex did not influence alpha or beta diversity but may influence taxonomic differences.

**Figure 2:**
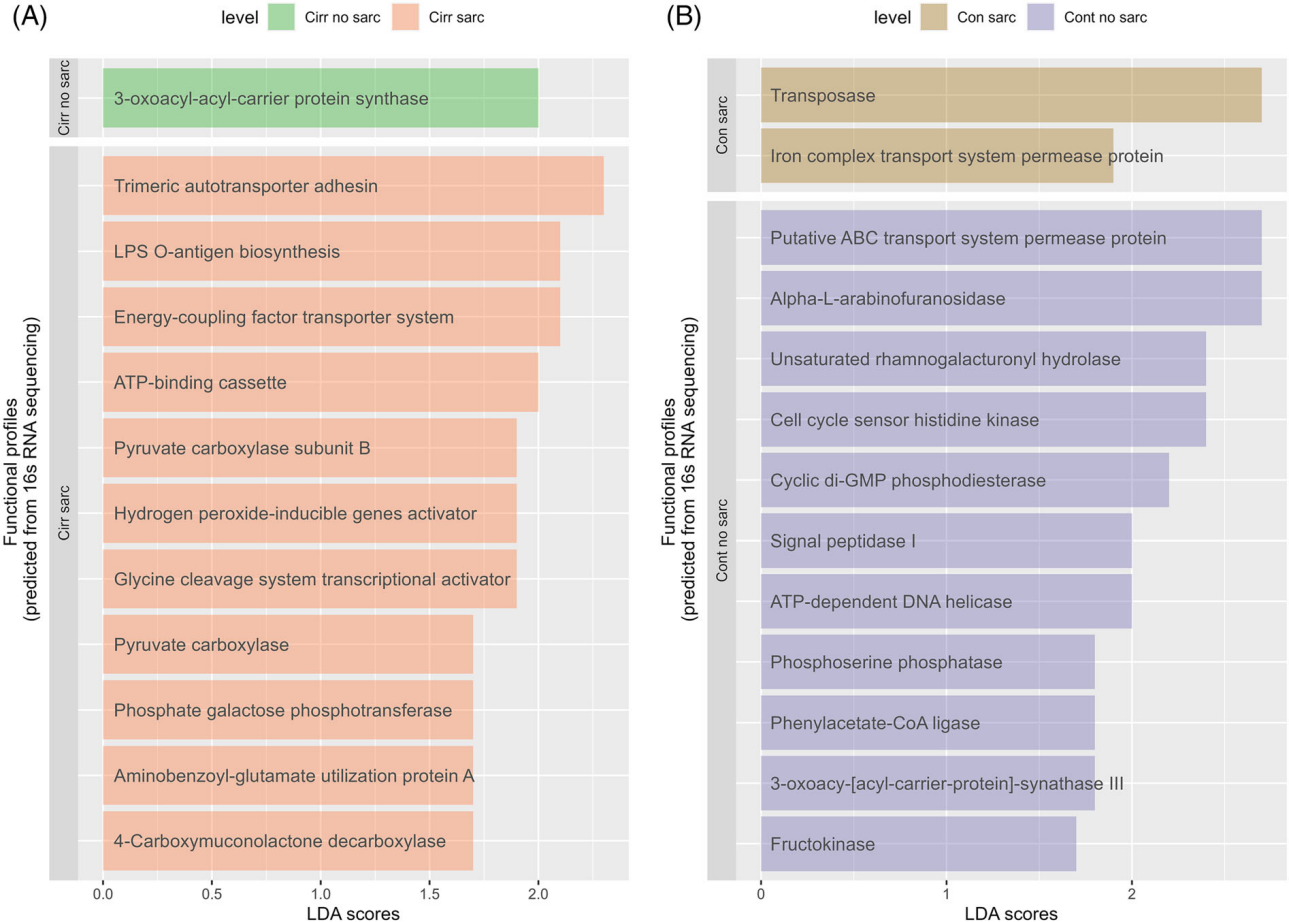
Tax4Fun‐predicted functional profiles between cirrhotic patients with and without sarcopenia (A) and controls with and without sarcopenia (B). LPS, lipopolysaccharide.

We conducted a functional prediction analysis to examine the changes in the gut microbiome composition associated with functional and metabolic pathway changes in cirrhotic patients with and without sarcopenia and non‐cirrhotic controls with and without sarcopenia. Using Tax4Fun, we identified significant differences in functional profiles between these groups. In cirrhotic patients with sarcopenia, the functional profiles were mainly related to the bacterial LPS O‐antigen biosynthesis pathway, hypermetabolic pathways and transporter systems. Specifically, we observed changes in pathways such as undecaprenyl‐phosphate galactose phosphotransferase, pyruvate carboxylase, pyruvate decarboxylase subunit B, 4‐carboxymuconolactone decarboxylase and aminobenzoic‐glutamate utilization protein A. On the other hand, in cirrhotic patients without sarcopenia, an association with the functional profile related to fatty acid biosynthesis (3‐oxoacyl‐[acyl‐carrier‐protein] synthase) was observed. Similarly, non‐cirrhotic controls with sarcopenia showed functional profiles associated with DNA transpose mechanisms (transposase) and transporters (iron complex transport systems permease protein). In contrast, non‐cirrhotic controls without sarcopenia were associated with functional profiles related to metabolism (alpha‐arabinofuranosidase, rhamnogalacturonyl hydrolase, phosphoserine phosphatase, phenylacetate‐CoA ligase and fructokinase), genetic information processing and cell division including cell cycle sensor histidine kinase, cyclic di‐GMP phosphodiesterase, ATP‐dependent DNA helicase and signal peptidase I (*Figure* *2*). These results suggest that sarcopenia is associated with reduced fatty acid biosynthesis, alterations in genetic information processing and cell division pathways regardless of cirrhosis. When both sexes were analysed separately, the gut microbiome functional profiles analysis showed no significant features associated with sarcopenia in cirrhosis and controls.

### Altered bile acid profiles, bile acid gene abundances and metabolomes in sarcopenia in cirrhosis

**Figure 3:**
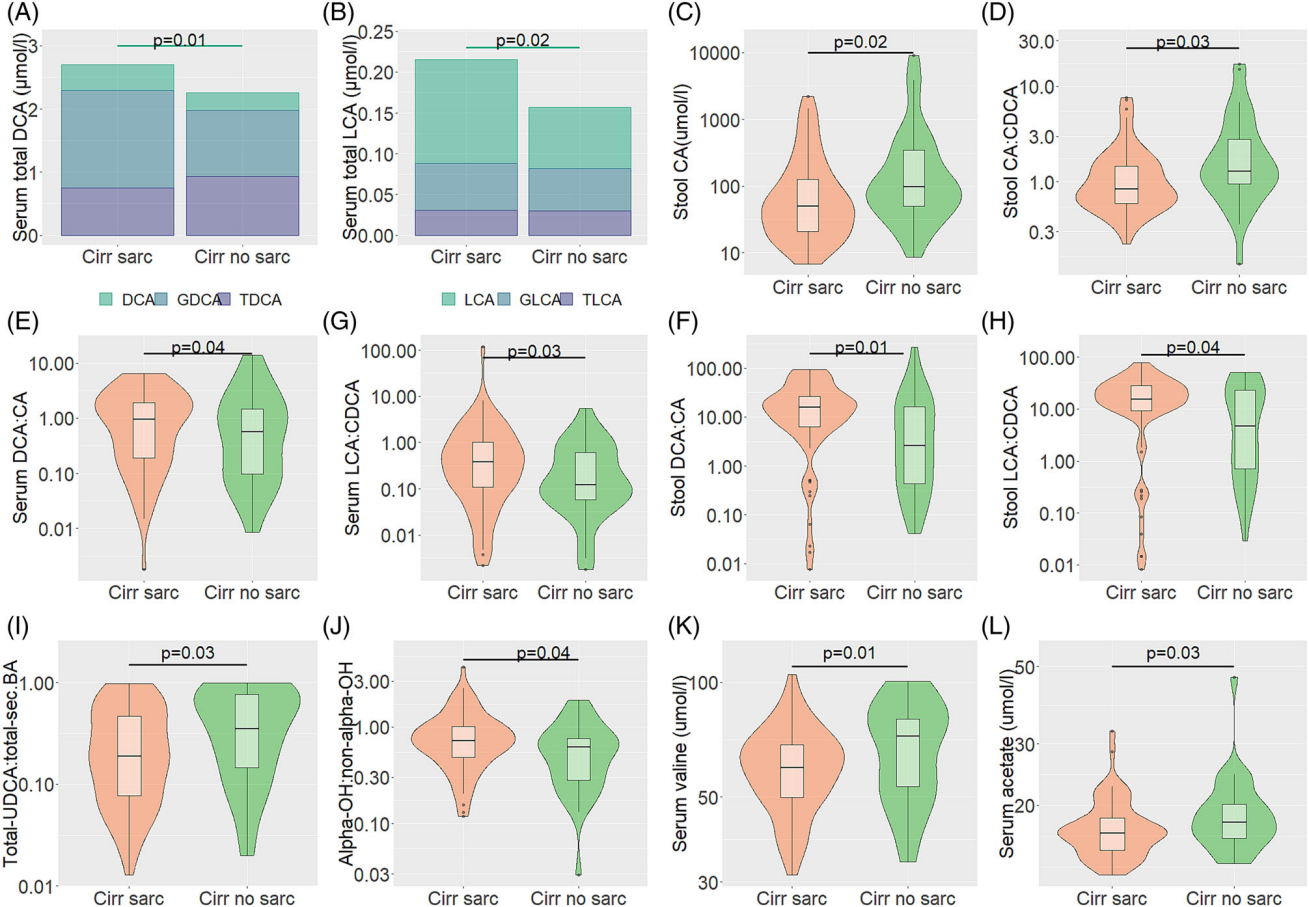
Altered secondary and primary bile acids (BAs) between cirrhotic patients with and without sarcopenia (A–C). Shift from the classical to the alternative pathways between cirrhotic patients with and without sarcopenia (D). Altered BA transformation from primary to secondary BAs between cirrhotic patients with and without sarcopenia (E–H). Altered BA pool hydrophilicity status between the cirrhotic patients with and without sarcopenia (I). Altered BA 12α‐hydroxylation between cirrhotic patients with and without sarcopenia (J). Altered gut microbiome‐derived metabolites, BCAA and short‐chain fatty acid between cirrhotic patients with and without sarcopenia (K, L).

In cirrhotic patients with sarcopenia, we observed significantly elevated serum secondary BAs (total DCA, total LCA, unconjugated DCA and unconjugated LCA) and stool total LCA compared with cirrhotic patients without sarcopenia (*P* = 0.04, *P* = 0.03, *P* = 0.01, *P* = 0.02 and *P* = 0.02, respectively; *Table* *2* and *Figure* *3* *A,B*). Conversely, stool CA and, therefore, stool CA:CDCA as a measure of a potential shift in BA synthesis from classical to alternative pathway were significantly reduced in cirrhotic patients with sarcopenia (*P* = 0.02 and *P* = 0.03, respectively; *Table* *3* and *Figure* *3* *C,D*). Additionally, the ratios of serum DCA:CA (*P* = 0.04), serum LCA:CDCA (*P* = 0.03), stool DCA:CA (*P* = 0.01) and stool LCA:CDCA (*P* = 0.04) were significantly elevated in cirrhotic patients with sarcopenia compared with cirrhotic patients without sarcopenia, indicating a possible alteration in gut microbiome composition characterized by an increase in the abundance of 7α‐dehydroxylating bacteria that may convert primary to secondary BAs (*Tables* *2* and *3* and *Figure* *3* *E–H*). Furthermore, cirrhotic patients with sarcopenia showed a significantly elevated ratio of 12 alpha‐hydroxylated to non‐12 alpha‐hydroxylated BAs (12‐α‐OH:non‐12‐α‐OH BAs) compared with cirrhotic patients without sarcopenia (*P* = 0.049; *Table* *2* and *Figure* *3* *J*), indicating increased BA 12α‐hydroxylation marked by increased CA and DCA. In contrast, the ratio of serum total ursodeoxycholic acid to total secondary BAs (T‐UDCA:total‐sec‐BAs) as a measure of BA pool hydrophilicity status was significantly reduced in cirrhotic patients with sarcopenia compared with cirrhotic patients without sarcopenia (*P* = 0.03; *Table* *2* and *Figure* *3* *I*). Contrary to our expectations, the ratios of secondary BAs conjugation, taurodeoxycholic acid to deoxycholic acid, glycodeoxycholic acid to deoxycholic acid, taurolithocholic acid to lithocholic acid, and glycolithocholic acid to lithocholic acid (TDCA:DCA, GDCA:DCA, TLCA:LCA and GLCA:LCA, respectively), were comparable between cirrhotic patients with and without sarcopenia (*P* > 0.05; *Tables* *2* and *3*), indicating that there were no abnormalities related to glycine or taurine conjugation between cirrhotic patients with and without sarcopenia. Furthermore, serum total DCA, total LCA, DCA, LCA, DCA:CA, LCA:CDCA, 12‐α‐OH:non‐12‐α‐OH BAs and T‐UDCA:total‐sec‐BAs, and stool total LCA, CA, CA:CDCA, DCA:CA and LCA:CDCA were comparable between controls with and without sarcopenia (*P* > 0.05). These results indicate that altered BA profiles are specific for sarcopenia in cirrhosis.

**Table 2:** Serum bile acid and bile acid ratio concentration of between‐groups comparison corrected for multiple testing

Bile acid profiles	Cirrhosis with sarcopenia (*n* = 78)	Cirrhosis without sarcopenia (*n* = 38)	Corrected *P*‐values
Total UDCA (μmol/L)	0.39 (0.27; 0.63)	0.68 (0.30; 2.31)	NS
GUDCA (μmol/L)	0.28 (0.17; 0.40)	0.39 (0.15; 1.15)	NS
TUDCA (μmol/L)	0.05 (0.01; 0.08)	0.12 (0.01; 0.47)	NS
UDCA (μmol/L)	0.09 (0.06; 0.14)	0.14 (0.11; 0.17)	NS
Total DCA (μmol/L)	1.58 (1.19; 2.45)	1.01 (0.45; 1.74)	0.04
GDCA (μmol/L)	0.81 (0.59; 1.15)	0.42 (0.14; 0.95)	NS
TDCA (μmol/L)	0.33 (0.18; 0.59)	0.20 (0.05; 0.49)	NS
DCA (μmol/L)	0.27 (0.21; 0.40)	0.07 (0.04; 0.27)	0.01
Total LCA (μmol/L)	0.14 (0.10; 0.19)	0.06 (0.03; 0.09)	0.03
GLCA (μmol/L)	0.02 (0.01; 0.04)	0.00 (0.00; 0.00)	NS
TLCA (μmol/L)	0.00 (0.00; 0.00)	0.00 (0.00; 0.00)	NS
LCA	0.09 (0.06; 0.10)	0.04 (0.03; 0.06)	0.02
Total CA (μmol/L)	7.89 (4.98; 15.22)	8.86 (5.48; 12.42)	NS
GCA (μmol/L)	4.48 (2.70; 6.63)	3.65 (2.79; 7.66)	NS
TCA (μmol/L)	2.25 (1.19; 4.51)	2.06 (0.92; 4.93)	NS
CA (μmol/L)	0.29 (0.24; 0.38)	0.32 (0.26; 0.53)	NS
Total CDCA	15.88 (9.84; 24.51)	21.13 (9.04; 32.61)	NS
TCDCA (μmol/L)	3.34 (2.29; 9.14)	7.97 (1.64; 13.49)	NS
GCDCA (μmol/L)	8.30 (6.24; 14.16)	11.68 (5.33; 16.97)	NS
CDCA (μmol/L)	0.34 (0.24; 0.50)	0.40 (0.26; 0.57)	NS
Shift from the classical to the alternative pathways			
CA:CDCA	1.28 (0.96; 1.88)	0.94 (0.70; 1.34)	NS
Gut microbiome modification			
DCA:CA	0.73 (0.34; 1.38)	0.14 (0.6; 0.84)	0.042
LCA:CDCA	0.25 (0.13; 0.46)	0.09 (0.04; 0.13)	0.028
UDCA:CDCA	0.26 (0.16; 0.34)	0.33 (0.14; 0.62)	NS
Conjugation of secondary bile acids			
TDCA:DCA	1.17 (0.53; 2.17)	1.09 (0.25; 2.67)	NS
GDCA:DCA	2.52 (1.82; 3.91)	3.04 (1.86; 4.04)	NS
GLCA:LCA	0.17 (0.08; 0.36)	0.00 (0.00; 0.00)	NS
TLCA:LCA	0.00 (0.00; 0.00)	0.00 (0.00; 0.00)	NS
Other bile acids			
T‐UDCA:total‐sec‐BAs	0.19 (0.14; 0.26)	0.35 (0.24; 0.65)	0.028
12α‐OH:non‐12α‐OH BAs	0.72 (0.64; 0.85)	0.62 (0.38; 0.73)	0.04
GLCA:CDCA	0.05 (0.00; 0.14)	0.00 (0.00; 0.00)	0.014
GDCA:CA	2.27 (1.39; 4.03)	0.91 (0.50; 2.22)	NS
TDCA:CA	0.75 (0.40; 1.42)	0.34 (0.17; 2.04)	NS
TLCA:CDCA	0.00 (0.00; 0.00)	0.00 (0.00; 0.00)	NS

*Note*: The data shown are absolute numbers and percentages, or median and 95% confidence interval (lower; upper). *P* < 0.05 values were obtained from the Student *t*‐test or Mann–Whitney *U* test and corrected using multiple testing corrections by the Benjamini–Hochberg method. Abbreviations: 12‐α‐OH:non‐12‐α‐OH BAs, 12 alpha‐hydroxylated to non‐12 alpha‐hydroxylated bile acids; CA, cholic acid; CA:CDCA, cholic acid to chenodeoxycholic acid; CDCA, chenodeoxycholic acid; DCA, deoxycholic acid; DCA:CA, deoxycholic acid to cholic acid; GCA, glycocholic acid; GCDCA, glycochenodeoxycholic acid; GDCA, glycodeoxycholic acid; GDCA:DCA, glycodeoxycholic acid to deoxycholic acid; GLCA, glycolithocholic acid; GLCA:LCA, glycolithocholic acid to lithocholic acid; GUDCA, glycoursodeoxycholic acid; LCA, lithocholic acid; LCA:CDCA, lithocholic acid to chenodeoxycholic acid; NS, not significant; TCA, taurocholic acid; TCDCA, taurochenodeoxycholic acid; TDCA, taurodeoxycholic acid; TDCA:DCA, taurodeoxycholic acid to deoxycholic acid; TLCA, taurolithocholic acid; TLCA:LCA, taurolithocholic acid to lithocholic acid; TUDCA, tauroursodeoxycholic acid; T‐UDCA:total‐sec‐BAs, total ursodeoxycholic acid to total secondary bile acids; UDCA, ursodeoxycholic acid; UDCA:CDCA, ursodeoxycholic acid to chenodeoxycholic acid.

**Table 3:** Stool bile acid and bile acid ratio concentration of between‐groups comparison corrected for multiple testing

Bile acid profiles	Cirrhosis with sarcopenia (*n* = 78)	Cirrhosis without sarcopenia (*n* = 38)	Corrected *P*‐values
Total UDCA (μmol/L)	18.41 (15.06; 30.80)	27.28 (14.90; 74.60)	NS
GUDCA (μmol/L)	2.35 (1.80; 3.00)	1.65 (1.30; 3.50)	NS
TUDCA (μmol/L)	0.81 (0.70; 1.41)	1.00 (0.80; 1.37)	NS
UDCA (μmol/L)	13.96 (10.10; 23.00)	15.80 (11.41; 56.90)	NS
Total DCA (μmol/L)	650.10 (418.30; 946.10)	364 (133.40; 691.60)	NS
GDCA (μmol/L)	9.05 (6.90; 11.70)	4.00 (1.70; 13.10)	NS
TDCA (μmol/L)	3.40 (2.40; 5.50)	1.400 (0.90; 7.24)	NS
DCA (μmol/L)	628.58 (402.80; 931.90)	323.70 (122.10; 684.80)	NS
Total LCA (μmol/L)	1221.20 (646.60; 1461.00)	430.91 (88.10; 1095.00)	0.01
GLCA (μmol/L)	0.90 (0.70; 1.10)	0.87 (0.70; 1.80)	NS
TLCA (μmol/L)	0.70 (0.50; 1.00)	0.52 (0.23; 0.61)	NS
LCA	838.05 (493.10; 1217.60)	427.80 (103.90; 903.39)	NS
Total CA (μmol/L)	80.65 (59.00; 97.00)	121.47 (82.20; 250.75)	NS
GCA (μmol/L)	10.30 (8.58; 17.50)	10.60 (5.50; 27.20)	NS
TCA (μmol/L)	8.20 (6.00; 14.40)	8.00 (5.00; 12.70)	NS
CA (μmol/L)	50.25 (34.30; 62.00)	98.30 (64.70; 148.30)	0.02
Total CDCA (μmol/L)	118.65 (78.70; 138.70)	123.19 (80.30; 240.29)	NS
GCDCA (μmol/L)	33.25 (22.10; 52.70)	26.30 (21.31; 47.70)	NS
TCDCA (μmol/L)	13.60 (7.80; 19.70)	12.30 (8.70; 19.20)	NS
CDCA (μmol/L)	46.80 (40.30; 65.20)	81.10 (50.30; 124.32)	NS
Shift from the classical to the alternative pathways			
CA:CDCA	0.84 (0.75; 1.11)	1.29 (1.04; 2.09)	0.028
Gut microbiome modification			
DCA:CA	15.96 (9.35; 18.13)	2.58 (0.86; 13.91)	0.014
LCA:CDCA	15.47 (12.67; 20.93)	4.65 (1.49; 21.31)	0.042
UDCA:CDCA	0.31 (0.24; 0.44)	0.29 (0.20; 0.56)	NS
Conjugation of secondary bile acids			
TDCA:DCA	0.01 (0.00; 0.01)	0.01 (0.01; 0.01)	NS
GDCA:DCA	0.01 (0.01; 0.02)	0.01 (0.01; 0.03)	NS
TLCA:LCA	0.00 (0.00; 0.00)	0.00 (0.00; 0.00)	NS
GLCA:LCA	0.00 (0.00; 0.00)	0.00 (0.00; 0.00)	NS
Other bile acids			
T‐UDCA:total‐sec‐BAs	0.01 (0.01; 0.02)	0.03 (0.01; 0.06)	0.037
12α‐OH:non‐12α‐OH BAs	0.94 (0.70; 1.11)	1.05 (0.79; 1.63)	NS
GLCA:CDCA	0.02 (0.02; 0.03)	0.01 (0.01; 0.03)	NS
GDCA:CA	0.20 (0.16; 0.28)	0.06 (0.03; 0.14)	NS
TDCA:CA	0.08 (0.06; 0.11)	0.04 (0.02; 0.08)	NS
TLCA:CDCA	0.01 (0.01; 0.02)	0.01 (0.00; 0.03)	NS

*Note*: The data shown are absolute numbers and percentages, or median and 95% confidence interval (lower; upper). *P* < 0.05 values were obtained from the Student *t*‐test or Mann–Whitney *U* test and corrected using multiple testing adjusted by the Benjamini–Hochberg method. Abbreviations: 12‐α‐OH:non‐12‐α‐OH BAs, 12 alpha‐hydroxylated to non‐12 alpha‐hydroxylated bile acids; CA, cholic acid; CA:CDCA, cholic acid to chenodeoxycholic acid; CDCA, chenodeoxycholic acid; DCA, deoxycholic acid; DCA:CA, deoxycholic acid to cholic acid; GCA, glycocholic acid; GCDCA, glycochenodeoxycholic acid; GDCA, glycodeoxycholic acid; GDCA:DCA, glycodeoxycholic acid to deoxycholic acid; GLCA, glycolithocholic acid; GLCA:LCA, glycolithocholic acid to lithocholic acid; GUDCA, glycoursodeoxycholic acid; LCA, lithocholic acid; LCA:CDCA, lithocholic acid to chenodeoxycholic acid; NS, not significant; TCA, taurocholic acid; TCDCA, taurochenodeoxycholic acid; TDCA, taurodeoxycholic acid; TDCA:DCA, taurodeoxycholic acid to deoxycholic acid; TLCA, taurolithocholic acid; TLCA:LCA, taurolithocholic acid to lithocholic acid; TUDCA, tauroursodeoxycholic acid; T‐UDCA:total‐sec‐BAs, total ursodeoxycholic acid to total secondary bile acids; UDCA, ursodeoxycholic acid; UDCA:CDCA, ursodeoxycholic acid to chenodeoxycholic acid.

Despite alterations in BA composition in stool and serum, we could not observe any significant differences in bile salt hydrolase (BSH), BA inducer CD and E (baiCD and E), 3 alpha‐hydroxysteroid dehydrogenases (3α‐HSDH), 3 beta‐hydroxysteroid dehydrogenases (3β‐HSDH), 5‐alpha reductase (5 AR), 7 alpha‐hydroxysteroid dehydrogenases (7α‐HSDH), 7 beta‐hydroxysteroid dehydrogenases (7β‐HSDH) and 12 alpha‐hydroxysteroid dehydrogenases (12α‐HSDH) gene abundances between cirrhotic patients with and without sarcopenia (*P* > 0.05; *Table* *S6*).

When analysing male and female patients separately, DCA, LCA and LCA:CDCA were significantly increased in male cirrhotic patients with sarcopenia compared with those without sarcopenia (*P* = 0.04, *P* = 0.03 and *P* = 0.02, respectively). However, DCA, LCA and LCA:CDCA were comparable in female cirrhotic patients with and without sarcopenia (*P* > 0.05). Notably, total DCA, total LCA in serum, stool LCA, stool CA:CDCA, serum DCA:CA, serum LCA, stool DCA:CA, stool LCA:CDCA, T‐UDCA:total‐sec‐BAs and 12‐α‐OH:non‐12‐α‐OH BAs were comparable in a separate analysis of male and female cirrhotic patients with and without sarcopenia. Similarly, serum total DCA, total LCA, DCA, LCA, DCA:CA, LCA:CDCA, 12‐α‐OH:non‐12‐α‐OH BAs and T‐UDCA:total‐sec‐BAs, and stool total LCA, CA, CA:CDCA, DCA:CA and LCA:CDCA were comparable between controls with and without sarcopenia in both sexes (*P* > 0.05). These results indicate that DCA, LCA and LCA:CDCA are features for sarcopenia in males with cirrhosis but not in females with cirrhosis. Multivariate regression analysis showed that T‐UDCA:total‐sec‐BAs, 12‐α‐OH:non‐12‐α‐OH BAs and GLCA:CDCA in serum and stool CA:CDCA were independently associated with sarcopenia in cirrhosis even when adjusted for sex and model of end‐stage liver disease (MELD) (*P* = 0.04, *P* = 0.03, *P* = 0.03 and *P* = 0.04, respectively; *Table* *4*). Notably, serum secondary BAs (total DCA, total LCA, unconjugated DCA and unconjugated LCA), DCA:CA and LCA:CDCA in serum, and total LCA, CA, DCA:CA and LCA:CDCA in stool were not independently associated with sarcopenia after adjusting for sex and MELD (*Table* *4*). This result indicates that T‐UDCA:total‐sec‐BAs, 12‐α‐OH:non‐12‐α‐OH BAs and GLCA:CDCA in serum and stool CA:CDCA are good predictors of sarcopenia in cirrhosis, irrespective of sex.

**Table 4:** Models for predicting sarcopenia in cirrhosis adjusted for sex and model of end‐stage liver disease

Predictor variables	Coefficients	Odd ratios (95% CI)	Adjusted *P*‐values
Model 1			
BMI	−0.20	0.83 (0.75; 0.92)	0.0001
Sex	1.23	3.42 (1.29; 9.12)	0.01
MELD	0.02	1.02 (0.93; 1.11)	0.73
Model 2			
MAMC	−0.04	0.96 (0.95; 0.98)	0.0001
Sex	2.13	8.43 (2.66; 26.70)	0.0001
MELD	0.01	1.01 (0.92; 1.11)	0.79
Model 3			
Serum total DCA	0.02	1.02 (0.89; 1.17)	0.77
Sex	1.14	3.14 (1.28; 7.71)	0.01
MELD	0.03	1.03 (0.95; 1.12)	0.46
Model 4			
Serum DCA	0.79	2.20 (0.64; 7.58)	0.21
Sex	1.07	2.91 (1.18; 7.16)	0.02
MELD	0.04	1.04 (0.95; 1.13)	0.42
Model 5			
Serum total LCA	0.67	1.95 (0.32; 11.84)	0.47
Sex	1.10	3.00 (1.22; 7.41)	0.02
MELD	0.03	1.04 (0.95; 1.13)	0.42
Model 6			
Serum LCA	3.28	26.68 (0.44; 1636.50)	0.12
Sex	1.06	2.90 (1.18; 7.13)	0.02
MELD	0.04	1.04 (0.96; 1.13)	0.34
Model 7			
Serum DCA:CA	0.03	1.03 (0.84; 1.26)	0.76
Sex	1.17	3.22 (1.32; 7.83)	0.01
MELD	0.03	1.04 (0.95; 1.13)	0.43
Model 8			
Serum LCA:CDCA	0.34	1.43 (0.88; 2.31)	0.15
Sex	1.12	3.05 (1.24; 7.53)	0.02
MELD	0.04	1.04 (0.96; 1.13)	0.35
Model 9			
Serum T‐UDCA:total‐sec‐BAs	−1.40	0.25 (0.06; 0.98)	0.04
Sex	0.94	2.57 (1.02; 6.48)	0.05
MELD	0.03	1.04 (0.95; 1.13)	0.43
Model 10			
Serum 12‐α‐OH:non‐12‐α‐OH BAs	0.93	2.54 (0.99; 6.55)	0.03
Sex	1.10	3.01 (1.21; 7.47)	0.02
MELD	0.04	1.04 (0.96; 6.55)	0.32
Model 11			
Serum GLCA:CDCA	2.71	15.12 (1.26; 180.91)	0.03
Sex	0.92	2.51 (1.01; 6.25)	0.05
MELD	0.04	1.04 (0.96; 1.13)	0.35
Model 13			
Stool total LCA	0.00	1.00 (1.00; 1.00)	0.84
Sex	1.56	4.75 (1.78; 12.71)	0.01
MELD	0.06	1.07 (0.97; 1.17)	0.18
Model 14			
Stool CA	0.00	1.00 (0.99; 1.00)	0.21
Sex	1.50	4.49 (1.65; 12.21)	0.01
MELD	0.06	1.06 (0.97; 1.17)	0.22
Model 15			
Stool CA:CDCA	−0.24	0.79 (0.62; 0.99)	0.04
Sex	1.50	4.19 (1.52; 11.54)	0.01
MELD	0.05	1.05 (0.96; 1.16)	0.29
Model 16			
Stool DCA:CA	0.01	1.00 (0.99; 1.02)	0.52
Sex	1.57	4.81 (1.79; 12.93)	0.01
MELD	0.06	1.06 (0.96; 1.17)	0.22
Model 17			
Stool LCA:CDCA	0.03	1.03 (0.97; 1.18)	0.07
Sex	1.53	4.63 (1.69; 12.68)	0.01
MELD	0.07	1.07 (0.97; 1.18)	0.16
Model 18			
Serum valine	−0.05	0.95 (0.92; 1.00)	0.01
Sex	1.68	5.38 (1.90; 15.22)	0.01
MELD	−0.05	0.95 (0.85; 1.06)	0.34
Model 19			
Serum acetate	−0.1	0.91 (0.82; 1.01)	0.09
Sex	1.38	3.98 (1.50; 10.48)	0.01
MELD	0.02	1.02 (0.93; 1.12)	0.66
Model 20			
Urine acetone	−0.04	0.96 (0.91; 1.02)	0.21
Sex	1.22	3.38 (1.23; 9.27)	0.02
MELD	0.05	1.05 (0.95; 1.15)	0.35

*Note*: All values were reported as coefficients and odds ratios (95% CI). Adjusted *P*‐values were obtained after multivariate logistic regression analysis to adjust for sex and MELD. Abbreviations: 12‐α‐OH:non‐12‐α‐OH BAs, 12 alpha‐hydroxylated to non‐12 alpha‐hydroxylated bile acids; BMI, body mass index; CA, cholic acid; CA:CDCA, cholic acid to chenodeoxycholic acid; CDCA, chenodeoxycholic acid; CI, confidence interval; DCA, deoxycholic acid; DCA:CA, deoxycholic acid to cholic acid; GLCA:CDCA, glycolithocholic acid to chenodeoxycholic acid; LCA, lithocholic acid; LCA:CDCA, lithocholic acid to chenodeoxycholic acid; MAMC, mid‐arm muscle circumference; MELD, model of end‐stage liver disease; T‐UDCA:total‐sec‐BAs, total ursodeoxycholic acid to total secondary bile acids.

In addition to altered BA profiles, untargeted NMR metabolomics analysis revealed valine and acetate in serum to be reduced in cirrhotic patients with sarcopenia compared with cirrhotic patients without sarcopenia (*P* = 0.01 and *P* = 0.03, respectively; *Table* *S7*). Furthermore, valine and acetate in serum and urine acetone were comparable among the controls with and without sarcopenia (*P* > 0.05), indicating that the altered metabolites were cirrhosis‐associated features of sarcopenia. We observed no significant difference in other stool and urine metabolites between cirrhotic patients with and without sarcopenia (*P* > 0.05). Only valine in serum stayed significantly different between male cirrhotic patients with and without sarcopenia when both sexes were analysed separately. Furthermore, multivariate regression analysis showed that serum valine was independently associated with sarcopenia in liver cirrhosis even after adjusting for sex and MELD.

### Systems biology analysis

To identify gut microbiome features, BAs and metabolomes associated with sarcopenia in liver cirrhosis, we applied LASSO regression and multiple logistic regression; all results are shown in *Table* *S8* and *Figure* *S3*. LASSO regression identified BMI, MAMC, albumin, irisin, creatinine, haematocrit, myostatin, C‐reactive protein (CRP), FGF‐21, sCD14, DAO, bilirubin, ALT, activated partial thromboplastin time (APTT), CA:CDCA, GDCA:CA, TDCA:CA, GLCA:CDCA, GDCA:DCA, TDCA:DCA, GLCA:LCA, TLCA:LCA, T‐UDCA:total‐sec‐BAs, 12‐α‐OH:non‐12‐α‐OH BAs, valine, phenylalanine, alanine and acetate to be associated with sarcopenia in cirrhosis. Furthermore, multivariate logistic regression confirmed BMI (*P* = 0.001), MAMC (*P* = 0.001), 12‐α‐OH:non‐12‐α‐OH BAs (*P* = 0.03), T‐UDCA:total‐sec‐BAs (*P* = 0.04), stool CA:CDCA (*P* = 0.04) and serum valine (*P* = 0.04) to be independently associated with sarcopenia in cirrhosis even when corrected for sex and the severity of the liver disease (*Table* *S8*).

### Metabolism pathways associated with sarcopenia in liver cirrhosis

**Figure 4:**
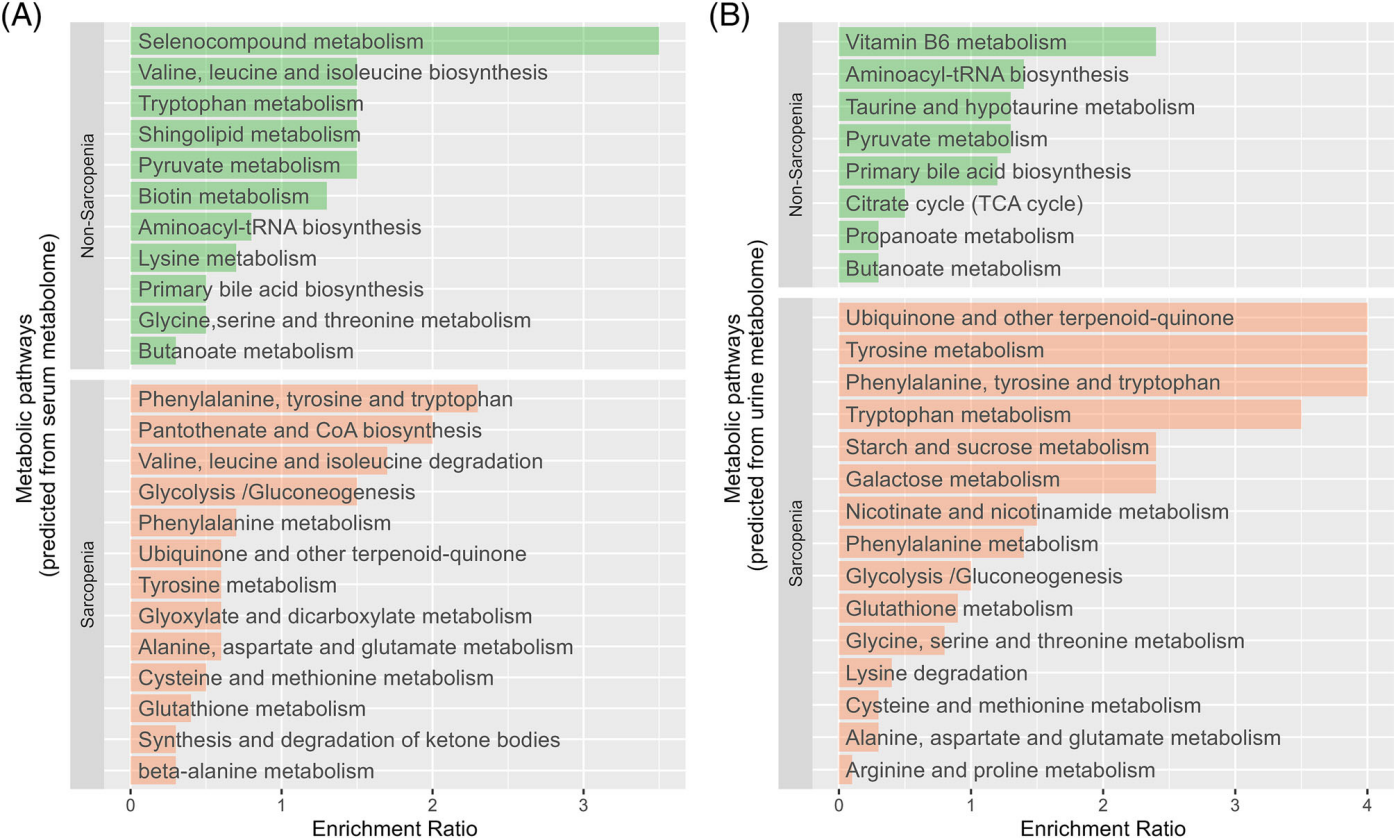
(A) Altered serum metabolite sets and associated pathways, and (B) urine metabolite sets and associated pathways between cirrhotic patients with and without sarcopenia.

As an explorative analysis of metabolic pathways associated with sarcopenia in liver cirrhosis, MSEA and pathway analysis were conducted; all the pathways with (*P* < 0.05) were considered significant (*Figure* *4* *A,B*). Ubiquinone biosynthesis; phenylalanine, tyrosine and tryptophan; and BCAA (valine, leucine and isoleucine) degradation (*P* = 0.04, *P* = 0.04 and *P* = 0.03, respectively) were the top pathways associated with cirrhotic patients with sarcopenia, whereas selenocompound metabolism was associated with cirrhotic patients without sarcopenia (*P* = 0.05). Additionally, BCAA (valine, leucine and isoleucine) degradation was associated with controls with sarcopenia (*P* = 0.02). These results indicate that BCAA degradation is a feature of sarcopenia irrespective of cirrhosis. BCAA (valine, isoleucine and alanine) degradation and the selenocompound pathways stayed associated with sarcopenia in both cirrhotic patients and controls when both sexes were analysed separately. These results indicate that sex did not influence the BCAA pathway degradation.

### Hierarchical clustering and correlational analysis of features associated with sarcopenia

We observed four hierarchical clusters indicated by four differently coloured dendrograms (*Figure* *S4*). Bacterial species linked to cirrhosis with sarcopenia including *B. fragilis* , *B. marseille* and *Sutterella* spp. were clustered together and correlated negatively with BMI, muscle mass and MAMC but showed a positive correlation with secondary BAs (DCA, LCA, total DCA and total LCA) and BA ratios (DCA:CA, LCA:CDCA and 12‐α‐OH:non‐12‐α‐OH BAs). These results suggest that *B. fragilis* , *B. marseille* and *Sutterella* spp. may individually or collectively play a potential role in the transformation of primary to secondary BAs. Notably, *V. parvula* , which is also associated with cirrhosis with sarcopenia, was clustered differently but equally showed a negative correlation with BMI, muscle mass and MAMC. *B. ovatus*, which is associated with cirrhosis without sarcopenia, showed a positive correlation with BMI and muscle mass, valine and acetate. Additionally, secondary BAs (DCA, LCA, total DCA and total LCA) and BA ratios (DCA:CA, LCA:CDCA and 12‐α‐OH:non‐12‐α‐OH BAs) were clustered together and showed a negative correlation with BMI, muscle mass and MAMC. These results further strengthen the notion that secondary BAs (DCA and LCA) may negatively impact muscle health. *B. fragilis* , *B. marseille* and *Sutterella* spp. consistently formed a distinct cluster. This cluster exhibited a consistent negative correlation with BMI, muscle mass and MAMC. Moreover, there was a positive correlation between this cluster and secondary BAs (DCA and LCA) and BA ratios (DCA:CA and 12‐α‐OH:non‐12‐α‐OH BAs), when both sexes were analysed separately. These findings indicate that these biomarkers are associated with sarcopenia, irrespective of sex.

## Discussion

Sarcopenia is a progressive and generalized skeletal muscle disorder that may be mediated by different factors including systemic inflammation, altered hormonal and myokine status, BAs and metabolomes.^7^ However, the molecular mechanism mediating the pathogenesis of sarcopenia in cirrhosis is not fully understood yet and therapeutic intervention is lacking. Identification of specific molecules mediating mechanisms underlying the gut–liver–muscle axis may result in the identification of novel therapeutic targets to treat loss of skeletal muscle mass and function in cirrhosis.

We conducted a prospective cohort study to elucidate the association of biomarkers reflecting the gut–liver axis with sarcopenia status in cirrhosis. Our results showed that BMI, muscle mass, MAMC, haematocrit and ALT significantly differed between cirrhotic patients with and without sarcopenia. We could further associate gut microbiome taxonomic composition to sarcopenia in cirrhosis. Particularly, *B. fragilis* , *B. marseille*, *Sutterella* spp. and *V. parvula* were associated with cirrhotic patients with sarcopenia and further correlated negatively with BMI, muscle mass and MAMC. *B. fragilis*, *Sutterella* spp. and *V. parvula* have been shown to produce LPS that may induce the production of pro‐inflammatory cytokines including tumour necrosis factor alpha (TNF‐α)‐mediated inflammation associated with loss of muscle mass.^19^, ^21^, ^22^ Age‐related sarcopenia has been associated with an increased abundance of *B. fragilis* and *Sutterella* spp.^22^ Furthermore, TNF‐α‐mediated inflammation has been associated with increased gut permeability and translocation of LPS mediating loss of muscle mass.^7^ Further along this line, we found that the gut microbiomes of cirrhotic patients with sarcopenia showed an enriched LPS antigen biogenesis pathway, which may be explained by the increase in the abundance of the potential LPS‐producing bacterial species in cirrhotic patients with sarcopenia. Notably, the design of our study does not allow to demonstrate causality.

Conversely, we showed *B. ovatus* to be more abundant in cirrhotic patients without sarcopenia and to correlate positively with muscle mass, MAMC, valine and acetate. Furthermore, *B. ovatus* independently predicted sarcopenia even after adjusting for drug use and the severity of the liver disease. The association of *B. ovatus* with better muscle health led us to hypothesize that this bacterial species may positively impact skeletal muscle health. Indeed, *B. ovatus* , *Bacteroides dorei* , *Bacteroides uniformis* and *Bacteroides vulgatus* have been shown to possess a conserved ability to inhibit LPS‐mediated inflammation.^23^, ^24^ Contrary to our expectations, although we found an alteration in gut microbiome composition, we did not find an indication of increased gut permeability, inflammation or altered hormonal/myokine status in cirrhotic patients with sarcopenia compared with cirrhotic patients without sarcopenia. However, zonulin, calprotectin and CRP were significantly elevated and IGF‐1 and irisin were significantly reduced in cirrhotic patients with sarcopenia, compared with non‐cirrhotic patients with and without sarcopenia. Additionally, myostatin has been shown to negatively regulate skeletal muscle mass through the inhibition of myoblast proliferation and differentiation.^25^ In humans, myostatin antibody treatment increased lean mass and muscle power in elderly persons.^26^ Skeletal muscle cells also secrete FGF‐21, which regulates skeletal muscle glucose uptake and muscle mitochondrial functions.^25^ Further, skeletal muscle cells secrete irisin in response to increased muscle protein degradation and myogenic differentiation.^25^ Irisin regulates skeletal muscle cells energetics associated with increased energy expenditure and glucose uptake.^25^ In humans, a significant reduction in irisin levels has been associated with sarcopenia in liver cirrhosis.^27^

Besides causing inflammation, the gut microbiome has a large metabolic capacity. One of these metabolic functions is the transformation of primary BAs into secondary BAs.^8^ During BA transformation, the critical steps include deconjugation by the action of BSH and 7α‐dehydroxylation through 7α‐HSDH.^28^, ^29^, ^30^ BSH activity is highly enriched in the bacterial genera *Lactobacilli*, *Bifidobacterium*, *Clostridium* and *Bacteroides*, whereas 7α‐dehydroxylation is enriched in a few bacterial genera, *Clostridium*, *Eubacterium* and *Blautia*.^31^ Considering this, the alteration of the gut microbiome composition in cirrhotic patients with and without sarcopenia prompted us to assess the possible gut microbiome‐associated alteration of BAs in our cohort. Even though BA synthesis and metabolism have a compensatory self‐regulatory mechanism through negative feedback,^8^ our results showed significantly reduced primary BAs (CA) in stool, elevated secondary BAs (DCA and LCA) in serum and, therefore, altered serum BA ratios (DCA:CA, LCA:CDCA and 12‐α‐OH:non‐12‐α‐OH BAs) in cirrhotic patients with sarcopenia compared with cirrhotic patients without sarcopenia. These results suggest a potential alteration in the gut microbiome composition marked with increased 7α‐dehydroxylating activity, leading to increased DCA and LCA in cirrhotic patients with sarcopenia in stool and serum. Consistent with our findings, a positive correlation between *Blautia* and DCA and the ratio of LCA:CDCA has been demonstrated, indicating the potential role of *Blautia* spp. as 7α‐dehydroxylating bacteria.^10^ We further demonstrated that DCA and LCA correlated positively with bacteria linked to sarcopenia in cirrhosis, notably, *B. fragilis* , *B. marseille* and *Sutterella* spp. This result lets us speculate that the interaction between *B. fragilis* , *B. marseille* and *Sutterella* spp. individually or collectively with BAs may result in altered BA profiles in cirrhotic patients with sarcopenia.

The possible crosstalk between the BAs and skeletal muscle is dependent on the activation of BA receptors.^32^ Indeed, CA, DCA and LCA are hydrophobic and cytotoxic, and potent agonists for BA receptor TGR5.^32^ TGR5 activated by DCA, LCA and CA has been shown to result in skeletal muscle protein breakdown, loss of muscle mass and skeletal muscle mitochondrial dysfunction in mice.^32^ Similarly, glycodeoxycholic acid (GDCA) and taurodeoxycholic acid (TDCA) have been associated with reduced skeletal muscle volume in non‐alcoholic fatty liver disease patients.^33^ We further showed that DCA and LCA correlated negatively with muscle mass and MAMC, indicating that DCA and LCA may potentially mediate the loss of muscle mass. Consistent with our result, DCA negatively correlated with upper and lower limb skeletal muscle volume in non‐alcoholic fatty liver disease patients.^33^ Contrary to our findings, LCA correlated positively with muscle mass in chronic liver disease.^34^ Differences in patient selection and ethnicity may explain this apparent contradiction.

Besides producing cytotoxic secondary BAs, DCA and LCA,^8^ the gut microbiome also produces hydrophilic and less cytotoxic secondary BAs, such as UDCA, which is regarded as a beneficial BA.^31^ Indeed, UDCA is produced by a few 7β‐HSDH‐producing bacteria from the genera *Clostridium*, *Eubacterium* and *Ruminococcus*.^8^ In the present study, our results showed that the ratio of T‐UDCA:total‐sec‐BAs was significantly higher in cirrhotic patients without sarcopenia compared with cirrhotic patients with sarcopenia. Additionally, T‐UDCA:total‐sec‐BAs independently predicted sarcopenia even after adjusting for the severity of the liver disease. We, therefore, hypothesize that microbiomes with a higher 7β‐HSDH activity may lead to increased production of UDCA in cirrhotic patients, which might protect from sarcopenia. In primary biliary cirrhosis, an increased abundance of UDCA has been shown to confer beneficial effects.^35^ Contrary to our expectation, in the current study, the abundances of BSH, 7α‐HSDH and 7β‐HSDH were comparable between cirrhotic patients with and without sarcopenia. However, the gene abundance finding may not be conclusive and limit our understanding of BA metabolism because gene abundance does not directly translate to gene expression. Additionally, BA profiles were comparable among the controls with and without sarcopenia, indicating that the observed alteration in the BA profile is a cirrhotic‐associated feature for sarcopenia. Considering these results, we hypothesize that the gut microbiome‐derived secondary BAs DCA and LCA and total UDCA may negatively and positively impact skeletal muscle health in cirrhosis, respectively. However, future in vitro studies using human skeletal muscle cell line are required to elucidate the possible mechanistic interplay between the DCA, LCA, UDCA and human skeletal muscle.

We observed significantly reduced valine levels in serum in cirrhotic patients with sarcopenia compared with cirrhotic patients without sarcopenia. Furthermore, metabolomics pathway analysis results showed that BCAA (valine, leucine and isoleucine) degradation was associated with cirrhotic patients with sarcopenia. Valine correlated positively with BMI, muscle mass, MAMC and *B. ovatus* . Multivariate regression further showed that serum valine was an independent predictor of sarcopenia in liver cirrhosis even when we corrected for the severity of the liver disease. Consistent with our findings, the levels of serum BCAA are significantly reduced in cirrhotic patients with sarcopenia.^36^ Additionally, BCAA supplementation improved muscle mass, and muscle strength, and increased skeletal protein synthesis in cirrhotic patients with sarcopenia.^37^ In an in vitro gut model, *B. ovatus* and valine production correlated positively.^9^ These results indicate that valine may positively impact skeletal muscle health and that *B. ovatus* may be critical for the bioavailability of valine in cirrhotic patients without sarcopenia.

Liver cirrhosis is a disease with male predominance, and sarcopenia also more common in males than in females with cirrhosis.^38^ Therefore, sex is an important confounder in our analysis. In our sex‐specific sensitivity analysis, we demonstrated that BMI, muscle mass and MAMC maintain their association with sarcopenia in both cirrhotic patients and controls in both sexes whereas the differences in microbiome composition showed sex specificity. Furthermore, secondary BAs DCA and LCA, and serum valine were significantly different only in male cirrhotic patients with and without sarcopenia, which was also observed in other disease cohorts, as males had significantly elevated levels of secondary BAs compared with females in colorectal cancer patients.^39^ The observed lack of significance for many parameters in separate analyses could be influenced by sample size limitations, reducing statistical power to detect significant effects in each sex group. Potential sex‐specific factors, such as hormonal influences or different body composition patterns, could also contribute to the observed sex differences.^12^ Consequently, our results should be interpreted with caution when generalizing the results to both sexes collectively. Future studies, specifically analysing female cirrhotic patients with and without sarcopenia, are therefore necessary to understand the potential sex‐specific factors that may contribute to the development of sarcopenia in females with cirrhosis to gain a more comprehensive understanding of the condition and its predictors.

Our study has some limitations: First, all the study participants were Caucasians, which limits the generalization of our findings to other ethnicities, considering that gut microbiome diversity and composition may depend on factors such as ethnicity, region and diet, among others.^40^ In this study, convenience sampling was used, and we acknowledge the potential bias related to it. The sex imbalance in our study population, which is inherit to the disease cohort we studied, as cirrhosis is a male‐dominated disease,^38^ may potentially lead to bias and influence the results. To avoid disadvantages for the underrepresented group (in our case, female participants), sex‐specific analysis and interpretation of results are important. Furthermore, our results should be interpreted cautiously because the observed associations do not allow to conclude causality; this would require further, mechanistic studies. Additionally, our findings were based on gene abundance rather than gene expression, limiting our understanding of gene functions.

In conclusion, our study demonstrated gut microbiome–host associations with altered gut microbiome composition, altered BA profiles and reduced serum valine in sarcopenic cirrhotic patients pointing towards a potential cirrhosis‐specific mechanistic interplay in understanding sarcopenia pathogenesis in cirrhosis. Our insights into sex‐specific associations further pave the way for future research to develop targeted interventions for managing sarcopenia in cirrhotic patients also considering sex‐specific peculiarities.

## Conflict of interest statement

Benard Aliwa, Angela Horvath, Julia Traub, Nicole Feldbacher, Hansjörg Habisch, Günter Fauler, Tobias Madl and Vanessa Stadlbauer declare that they have no conflict of interest.

## Supporting information


**Table S1.** Drugs used by cirrhotic patients with and without sarcopenia.
**Table S2.** Primer sequence for bile acid genes.
**Table S3.** Clinical and demographic characteristics of control group comparison. Median (95% CI).
**Table S4.** Characteristics of male cirrhotic patients with and without sarcopenia. Median (95% CI).
**Table S5.** Characteristics of female cirrhotic patients with and without sarcopenia. Median (95% CI).
**Table S6.** Bile acid and acetate gene abundance distribution between groups. Median (95% CI).
**Table S7.** Serum, stool, and urine metabolite concentrations between groups adjusted for multiple testing. Median (95% CI).
**Table S8.** Models for predicting sarcopenia in cirrhosis adjusted for MELD and drug use. All values were reported as coefficients and odds ratios (95% CI).
**Figure S1.** Altered anthropometric measurements (A) and altered muscle mass biomarkers (B‐C) between cirrhotic patients with and without sarcopenia and controls with and without sarcopenia. Altered laboratory markers between cirrhotic patients with and without sarcopenia (D‐F).
**Figure S2.** Chao1 index as a measure of alpha diversity between male cirrhotic patients with and without sarcopenia (A). Chao1 index as a measure of alpha diversity between female cirrhotic patients with and without sarcopenia (B). Bray‐Curtis as a measure of beta‐diversity between male cirrhotic patients with and without sarcopenia (C). Bray‐Curtis as a measure of beta‐diversity between male cirrhotic patients with and without sarcopenia (D). Bacteria identified by regularized logistic least absolute shrinkage and selection operator (LEfSe) to be associated with male cirrhotic patients with and without sarcopenia (E). Bacteria identified by regularized logistic least absolute shrinkage and selection operator (LEfSe) to be associated with male cirrhotic patients with and without sarcopenia (F).
**Figure S3.** Features identified by LASSO regression as predictors of sarcopenia in liver cirrhosis.
**Figure S4.** Correlation between clinical parameters, bacterial OTUs, BAs, and metabolites. Green and Indian red indicate positive and negative correlations, respectively.
