# Supplementary material for: Altered gut microbiome, bile acid composition and metabolome in sarcopenia in liver cirrhosis
Source: J Cachexia Sarcopenia Muscle. 2023 Sep 28;14(6):2676–91. doi: 10.1002/jcsm.13342 (PMC10751428; doi:10.1002/jcsm.13342)
Supplement: Supplementary file 1 — Table S1. Drugs used by cirrhotic patients with and without sarcopenia. Table S2. Primer sequence for bile acid genes. Table S3. Clinical and demographic characteristics of control group comparison. Median (95% CI). Table S4. Characteristics of male cirrhotic patients with and without sarcopenia. Median (95% CI). Table S5. Characteristics of female cirrhotic patients with and without sarcopenia. Median (95% CI). Table S6. Bile acid and acetate gene abundance distribution between groups. Median (95% CI). Table S7. Serum, stool, and urine metabolite concentrations between groups adjusted for multiple testing. Median (95% CI). Table S8. Models for predicting sarcopenia in cirrhosis adjusted for MELD and drug use. All values were reported as coefficients and odds ratios (95% CI). Figure S1. Altered anthropometric measurements (A) and altered muscle mass biomarkers (B‐C) between cirrhotic patients with and without sarcopenia and controls with and without sarcopenia. Altered laboratory markers between cirrhotic patients with and without sarcopenia (D‐F). Figure S2. Chao1 index as a measure of alpha diversity between male cirrhotic patients with and without sarcopenia (A). Chao1 index as a measure of alpha diversity between female cirrhotic patients with and without sarcopenia (B). Bray‐Curtis as a measure of beta‐diversity between male cirrhotic patients with and without sarcopenia (C). Bray‐Curtis as a measure of beta‐diversity between male cirrhotic patients with and without sarcopenia (D). Bacteria identified by regularized logistic least absolute shrinkage and selection operator (LEfSe) to be associated with male cirrhotic patients with and without sarcopenia (E). Bacteria identified by regularized logistic least absolute shrinkage and selection operator (LEfSe) to be associated with male cirrhotic patients with and without sarcopenia (F). Figure S3. Features identified by LASSO regression as predictors of sarcopenia in liver cirrhosis. Figure S4. Correlation between [file JCSM-14-2676-s001.docx]

**Altered Gut Microbiome, Bile Acid Composition, and Metabolome in Sarcopenia in Liver Cirrhosis**

Benard Aliwa^1,7^, Angela Horvath^1,2^, Julia Traub^3^, Nicole Feldbacher^1^^,2^, Hansjörg Habisch^4^, Günter Fauler^6^, Tobias Madl^4,5^, Vanessa Stadlbauer^1,2*^

^1^*Division of Gastroenterology and Hepatology, Department of Internal Medicine, Medical University of Graz, Austria*

*^2^Centre for Biomarker Research in Medicine (CBmed), Graz, Austria*

*^3^Department of Clinical Medical Nutrition, University Hospital Graz, Graz, Austria*

*^4^Gottfried Schatz Research Center, Molecular Biology and Biochemistry, Medical University of Graz, Austria*

*^5^*BioTechMed-Graz, Graz, Austria

*^6^Clinical Institute for Medical and Chemical Laboratory Diagnostics, Graz, Austria*

*^7^Department of Food Science, Nutrition and Technology, University of Nairobi, Kenya*

Materials and methods

## DNA extraction and sequencing

Study participants were provided with sterile dry screw-top containers for collecting stool samples. Stool samples were received and frozen at -80 °C, waiting for further analysis. According to the manufacturer's instructions, total genomic DNA was extracted from stool samples using a MagnaPure LC DNA isolation kit (Roche, Mannheim, Germany). The V1-V2 hypervariable regions were specifically targeted for PCR amplification using the (primer sets forward-AGAGTTTGATCCTGGCTCAG and reverse-TGCTGCCTCCCGTAGGAGT). Samples were sequenced using an Illumina MiSeqTM technology (Illumina, Eindhoven, the Netherlands) as described before (1). These sequence data have been submitted to NCBI Sequencing Read Archive (accession number, PRJNA933898, <https://www.ncbi.nlm.nih.gov/sra/PRJNA933898>).

Raw sequence reads were pre-processed using QIIME 2 tools on a local Galaxy instance (<https://galaxy.medunigraz.at>) (2). Denoising (primers removing, quality filtering, correcting errors in marginal sequences, removing chimeric sequences, removing singletons, joining paired-end reads, and dereplication) was done with DADA2 as described before (3). Taxonomy was assigned based on the Silva V132 database release at 99% operational taxonomic unit level with a Naïve Bayes classifier (4). When necessary, sequences were blasted in the NCBI database for further classification (5). For the analysis, the OTU table in biom format and the metadata file of the study participants were uploaded into Calypso 8.84 (<http://cgenome.net/wiki/index.php/Calypso>) (6). The OTU table was filtered and normalized to remove Cyanobacteria and Chloroplasts, samples with less than 1000 sequence reads, and taxa with less than 0.01 relative abundance across all samples.

## ELISA

Commercially available ready-to-use solid-phase sandwich ELISA kits were used to quantify LBP (Hycult Biotechnology, Uden, Netherlands), CD14 (R&D Systems, Abingdon, UK), IGF-1 (Immundiagnostik, Germany), myostatin (Immundiagnostik, Germany), FGF-21 (BioVendor), irisin (BioVendor, Czech Republic), DAO (Immundiagnostik, Germany) in serum. Furthermore, ELISA kits were used to quantify zonulin (Immundiagnostik) and calprotectin (Immundiagnostik) in the stool. The manufacturer's protocol was duly followed during the analysis.

## Ultra-high-performance liquid chromatography-tandem mass spectrometry (UPLC-MS/MS) for analysis of serum and fecal bile acids

Bile acids were quantified using ultra-high-performance liquid chromatography-tandem mass spectrometry (UPLC-MS/MS). Organic solvents (HPLC grade) for precipitation were obtained from (Sigma Aldrich Handels GesmbH, Vienna, Austria) and Merck (Germany), and bile acid controls (abnormal and normal) were supplied by Trinity Biotech (Ref B6021 and B5021). All bile acid standards were purchased from (Sigma Aldrich, Germany, and Cayman Chemicals in the USA). Bile acid standard stock solutions at a concentration of 2 mmol/L were prepared in high-grade methanol. Standards working solutions of different bile acids were prepared in high-grade methanol at a final 25 nmol/ml concentration. An internal standard (IS) at 2nmol/100µl methanol concentration was used. The calibration curve with a concentration range of 1-3000nmol/ml for BAs was obtained by mixing 50µl of each BAs standard stock with 950µl of methanol. The mixture was then serially diluted with methanol (1:1, v/v) up to the third tube. 100µl internal standard was added to each of the serial dilutions’ tubes. The mobile phase solvents were supplied by (Sigma Aldrich Handels GesmbH, Vienna, Austria).

For serum samples, 10μl of serum was deproteinized using 400 μl acetonitrile and centrifuged at 10,000g at 4 ºC for 12 min. Supernatant from the samples and controls was transferred into fresh tubes. 100 μl IS (2 nmol/100 μl in methanol) was added to each sample tube, and the mixture was homogenized by vortexing. Samples and controls were centrifuged for 10 minutes at 4000g at 4º C. 100 μl supernatant of the samples and controls were transferred into a fresh tube and dried under a stream of nitrogen and re-dissolved in 100 μl of mobile phase solvent B (MeOH and ammonium hydroxide, 100:0.1; v/v) and transferred to auto-sampler vials. The UHPLC-MS was conducted on 5 ml reversed-phase (Agilent, USA), using a methanol/water gradient. 10 μl was the injection volume of all samples and controls. The absolute concentrations of BAs were calculated using calibration curves generated by internal standards.

For stool samples, 10mg of dried stool was homogenized in 2 ml of NaOH (0.1M). The mixture was incubated at 60°C for 1 hr. This was followed by the mixture being homogenized in a sonicator water bath for 15 seconds. The stool homogenate was diluted with NaOH (1:100, 0.1M). After that, 100µl IS (2nmol/100µl methanol), and 4 ml of deionized water were added to the diluted stool samples. The suspension was sonicated in a water bath for a further 15 seconds. 4ml acetonitrile (80% v/v) was added to the sonicated samples suspension. The sample suspensions were incubated at room temperature for 20 minutes and centrifuged for 20 minutes at 2500 rpm. The supernatant was then collected and placed in clean tubes. The supernatant was dried overnight in a speed-vac (-110°C) under a stream of nitrogen. The overnight dried sample was dissolved in 4ml of ammonium acetate (15mM, PH=5.3). The preconditioning of a 10 ml reversed-phase cartridge C18 (Agilent, USA) was achieved by adding 5 ml of deionized water, 5 ml of methanol, and 5 ml of deionized water in that order. After that sample suspension was loaded into a preconditioned C18 cartridge. The washing involved 6 ml of water (HPLC high grade), 6 ml of hexane, and 6 ml of water (HPLC high-grade water). The C18 cartridge content was eluted into clean labelled tubes. The samples were eluted using 3ml methanol (HPLC gradient grade). The eluted samples were dried overnight in a stream of nitrogen using speedVacs (-110°C). After that 100µl of mobile phase B was added to the dried tube content and 100µl pipetted into autosampler vials. The sealed samples were stored at -20°C until measurements. 10 μl was the injection volume of all samples and controls (normal and abnormal). The absolute concentrations of BAs were calculated using calibration curves generated by internal standards. This protocol was partly adopted from (7, 8).

## Quantification of serum, stool, and urine metabolites using Nuclear Magnetic Resonance (NMR)

To remove protein and to quench enzymatic reaction, 200 µl of the samples (serum, urine, and stool) were mixed with 400 µl methanol and samples stored for 1 hour at -20 °C waiting further processing (9). The samples were then centrifuged at 17,949 rcf for 30 minutes at 4 °C. The quantification of serum, stool, and urine metabolites was performed using 310 K on NMR spectroscopy (Bruker Avance 600 MHz) equipped with TXI probe head and processed as described (10). The 1D CPMG (Carr-Purcell_Meiboom_Gill) pulse sequence (cpmgpr1d, 512 scans, 73,728 points in F1,11,904.76 HZ spectral width, 512 transients, recycle delay 4 s) with water suppression using pre-saturation, was used for ^1^H 1D NMR experiments. Samples were thawed at room temperature and mixed by vortexing. 200 μl of serum or stool was mixed with 400 μl of a buffer solution (15% D_2_O, 3mM trimethylsilypropanoic acid (TMSP), and 0.9% NaCl). Equally, 400 μl of the urine sample was mixed with 200 μl of PBS (0.5 M, PH=7.00, 33% D_2_O containing 3 mM NaN3 and 3 mM TSP). The mixtures were centrifuged at 12000g at 4 ºC for 10 min. 500 μl of the supernatant was transferred into an mm NMR tube for further analysis. (9). The serum, stool, and urine metabolomics quantification were conducted by Professor Tobias Madl’s Lab group.

## Bile acids gene abundance quantification by qPCR

To judge the theoretical potential expression of the gut microbiome-derived enzymes critical for BAs microbial transformation, we measured BAs gene abundances including bile salt hydrolase (BSH), bile acid inducer CD, and E (baiCD and E), 3 alpha-hydroxysteroid dehydrogenases (3α-HSDH), 3 beta-hydroxysteroid dehydrogenases (3β-HSDH), 5-alpha reductase, 7 alpha-hydroxysteroid dehydrogenases (7α-HSDH), 7 beta-hydroxysteroid dehydrogenases (7β-HSDH), and 12 alpha-hydroxysteroid dehydrogenases (12α-HSDH). The primer BLAST tool was used to obtain the primers sequences (11). The primers were synthesized and supplied by Eurofins Genomics (Vienna, Austria, Table S 2). The genomic DNA isolated from stool samples were used in the qPCR experiment to quantify BAs gene abundance. The qPCR was performed in Bio-Rand (Roche Diagnostic, Mannheim, Germany). 25µl reaction volume was used containing: 12.5 µl SYBR green (SYBR, Promega, Madison, WI USA) and 5.5 µl of RNA/DNases free water (Promega, Madison, WI USA), 1.0µl of 10.0 µM for forward primer and 1.0µl of 10.0 µM reverse primer (Eurofins Genomics, Vienna, Austria), and 5.0 µl of template DNA. For the standard curve, 5.0 µl DNA from each sample was pooled. 7 µl of the DNA was pipetted into a tube containing 14 µl of DNAse free water (1:3), this process was serially repeated until the 7^th^ tube to achieve desired varied concentrations used for the standard curve. The qPCR thermocycling conditions included: 95 ºC for 2 min followed by 40 cycles of denaturation at 98 ºC for 10 sec, primer-specific annealing at 52 ºC for 20 sec, and elongation at 68 ºC for 1 min. The BAs gene abundance was quantified relative to bacterial 16s rDNA genes. The normalized BAs gene abundance data was used for statistical analysis. The method was adopted from previously published protocols (12)

**Table** S 1**.** Drugs used by cirrhotic patients with and without sarcopenia

|  | **Cirr Sarcopenia (n=78)** | **Cirr non-Sarcopenia (n=38)** | **P-values** |
| --- | --- | --- | --- |
| **PPI use (n%)** | Yes 39 (50.6); No 39(39.8) | Yes19(24.7); No 19(19.4) | Ns |
| **Β-blockers** | Yes 49(59.8); No 29(31.2) | Yes 20(24.4); No 18(19.4) | Ns |
| **Laevolac** | Yes 17(70.8); No 61(40.4) | Yes 7(29.2); No 31(20.5) | Ns |
| **Statine** | Yes 6(28.6); No 72(46.8) | Yes 6(28.6); No 32(20.8) | Ns |
| **Diuretic** | Yes 47(71.2); No 31(28.4) | Yes 16(24.2); No 22(20.2) | Ns |
| **Antihypertensive** | Yes 16(31.4); No 62(50) | Yes 14(27.5); No 24(19.4) | Ns |
| **Opiate** | Yes 5(41.7); No 73(44.8) | Yes 3(25); No 35(21.5) | Ns |
| **Antidepressants** | Yes 16(42.1); No 62(45.3) | Yes 8(21.1); No 30(21.9) | Ns |
| **Laxatives** | Yes 5(50); No 73(44.4) | Yes 1(10); No 37(22.4) | Ns |
| **Sedatives** | Yes 15(68.2); No 63(41.2) | Yes 3(13.6); No 35(22.9) | Ns |
| **Immunosuppressive** | Yes 2(16.6); No 76(46.6) | Yes 3(25); No 35(21.5) | Ns |
| **Cholesterol-lowering** | Yes 0(0); No 78(45.6) | Yes 2(50); No 36(21.1) | Ns |
| **Testosterone** | Yes 1(100); No 77(44.3) | Yes 0(0); No 38(21.8) | Ns |
| **Antifungal** | Yes 1(100); No 77(44.3) | Yes 0(0); No 38(21.8) | Ns |
| **Botanical** | Yes 2(40); No 76(44.7) | Yes 1(20); No 37(21.8) | Ns |

*^1Note^*^: Abbreviations: PPI; proton pump inhibitor. P-values were determined via the Chi-Square test r^

**Table** S 2 Primer sequence for bile acid genes

| **Gene label** | **F/R** | **Primer sequence** | **Gene for** | **Product**  **Length** |
| --- | --- | --- | --- | --- |
| BSH | F | TGGCTAGCCTGCTTTTCTTG | Bile salt hydrolase | 318 bp |
|  | R | CCGTGGCAAAGTTATCAAGC |  |  |
| 3α-HSDH | F | AGAAGATCTACGCCGAAGCG | 3α-hydroxysteroid dehydrogenase | 159 bp |
|  | R | CCATGTACACCTTGGCACCT |  |  |
| 3β-HSDH | F | CAGGCTACCGCCACATAGAC | 3β-hydroxysteroid dehydrogenase | 140 bp |
|  | R | GGTGTCGTACCCTCTTTCCG |  |  |
| 5 AR | F | CCTAAGGAATCTCAGAAAACCAGG | 5α- reductase | 117 bp |
|  | R | GCATAGCCACACCACTCCATGA |  |  |
| BaiCD | F | CAGCCCRCAGATGTTCT TTG | 7α-dehydroxylase | 200 bp |
|  | R | GCATGGAATTCHACTGCRTC |  |  |
| BaiE | F | GCCCGAAGGAAGTTACCGAT | 7-alpha dehydratase | 93 bp |
|  | R | TGTCAATGGTGATCTCCGGC |  |  |
| 7α-HSDH | F | CACGGGACAAACAATCGTCG | 7α-hydroxysteroid dehydrogenase | 209 bp |
|  | R | CCACTCTTCCCCCTTTTCGAT |  |  |
| 7β-HSDH-F | F | TACGCGAGATCATCGAAGGC | 7β-hydroxysteroid dehydrogenases | 230 bp |
|  | R | TTCCGGGTACGGGAAGTAGT |  |  |
| 12α- HSDH | F | GGCTTCTGCGTCGGGTATT | 12α-hydroxysteroid dehydrogenase | 192 bp |
|  | R | GCATATGCACACTGACCGAAGT |  |  |
| 16s rRNA | F | GTGSTGCAYGGYTGTCGTCA |  | 527 bp |
|  | R | ACGTCRTCCMCACCTTCCTC |  |  |

*^2Note^*^: Abbreviations: BSH, bile salt hydrolase; BaiCD E bile acid inducer CD, and E; 3α-HSDH, 3 alpha-hydroxysteroid dehydrogenases; 3β-HSDH, 3 beta-hydroxysteroid dehydrogenases; 7α-HSDH, 7 alpha-hydroxysteroid dehydrogenases; 7β-HSDH, 7 beta-hydroxysteroid dehydrogenases; 12α-HSDH, 12 alpha-hydroxysteroid dehydrogenases^

**Table** S 3**.** Clinical and demographic characteristics of control group comparison**.** Median (95% CI).

|  | Control sarcopenia (n=39) | Control no sarcopenia (n=20) | P-values |  |
| --- | --- | --- | --- | --- |
| Sex (n/%) | Male 20 (51.3%); female  19 (48.7%) | Male 13 (65.0%); female  7 (35.0%) | Ns |  |
| Age (years) | 59 (56;66) | 59 (53;67) | Ns |  |
| Body mass index (kg/m^2^) | 23.9 (21.5;25.2) | 28.2 (26.0;29.7) | p<0.0001 |  |
| Charlson comorbidity index | 0 (0;0) | 1 (0;6) | Ns |  |
|  |  |  |  |  |
| Muscle mass and function | | | | |
| Muscle mass (cm^2^/m^2^) | 35.99 (33.36;42.88) | 54.48 (50.00;56.12) | p<0.0001 |  |
| Handgrip strength (kg) | 27.30 (23.30;36.00) | 39.39 (29.00;45.30) | Ns |  |
| Mid-arm muscle circumference (cm) | 24.49 (22.70;25.09) | 26.48 (25.11;28.90) | p=0.003 |  |
| Triceps Skinfold Thickness (mm) | 11.70 (9.50;16.00) | 15.00 (11.40;19.00) | Ns |  |
| Gait speed (m/sec) | 1.04 (0.93;1.11) | 1.02 (0.98;1.19) | Ns |  |
| Chair rise (sec) | 16.69 (14.72;19.37) | 15.54 (13.43;17.09) | Ns |  |
| Liver functions | | | | |
| Bilirubin (mg/dL) | 0.19 (0.16;0.24) | 0.18 (0.15;0.27) | Ns |  |
| Albumin (g/dL) | 4.4 (4.4;4.6) | 4.6 (4.4;4.8) | p=0.015 |  |
| Total protein (g/dL) | 7.3 (7.2;7.5) | 7.6 (7.4-7.7) | Ns |  |
| Prothrombin time (INR) | 0.92 (0.88;0.96) | 0.94 (0.89;0.98) | Ns |  |
| AST (U/L) | 22 (20;26) | 22 (19;28) | Ns |  |
| ALT (U/L) | 21 (20;33) | 26 (18;34) | Ns |  |
| GGT(U/L) | 32 (24;52) | 31 (22;55) | Ns |  |
| CRP (mg/dL) | 2.0 (1.0;3.9) | 4.2 (3.2;9.0) | Ns |  |
| Hematocrit (%) | 40.2 (38.5;41.2) | 42.8 (38.3;44.7) | Ns |  |
| Creatinine (mg/dL) | 0.81 (0.77;0.93) | 0.97 (0.81;1.01) | Ns |  |
| Zonulin (ng/mL) |  |  | Ns |  |
| DAO (U/ml) | 18.74 (16.93;21.59) | 13.45 (7.78;21.60) | Ns |  |
| LBP (µgml) | 16.04 (15.29;19.53) | 18.58 (16.08;21.16) | Ns |  |
| CD14 (µgml) | 1.66 (1.54;1.85) | 1.65 (1.56;1.87) | Ns |  |
| Calprotectin (μg/g) |  |  | Ns |  |
| Myostatin (ng/ml) | 38.59 (36.91;40;82) | 44.25 (36.21;50.08) | Ns |  |
| Irisin (µg/ml) | 2.58 (2.08;3.49) | 2.24 (1.85;2.98) | Ns |  |
| FGF21 (ng/ml) | 0.33 (0.25;0.42) | 0.26 (0.19;0.43) | Ns |  |
| IGF-1 (ng/ml) | 138.49 (126.64;167.24) | 116.43 (101.44;165.61) | Ns |  |

*^3Note^*^: The data shown are absolute numbers and percentages, or median and 95% confidence interval (lower; upper). P<0.05, values were obtained from the student’s t-test or Mann-Whitney U test.^

^Abbreviations: INR, international normalized ratios; AST, aspartate aminotransferase; ALT, alanine aminotransferase; GGT, gamma-glutamyl-transferase; CRP, C-reactive protein; DAO, diamino-oxidase; LBP, lipopolysaccharide; sCD14, soluble CD14; FGF-21, fibroblast growth factor 21; IGF-1, insulin-like growth factor 1.^

**Table** S 4. Characteristics of male cirrhotic patients with and without sarcopenia. Median (95% CI)

|  | **Cirrhosis sarcopenia (n=64)** | **Cirrhosis no sarcopenia (n=22)** | **P-values** |
| --- | --- | --- | --- |
|  |  |  |  |
| **Age (years)** | 64 (61;68) | 62 (60;67) | Ns |
| **Body mass index (kg/m^2^)** | 25.9 (24.2;27.3) | 28.7 (27.3;30.8) | 0.001 |
| **Charlson comorbidity index** | 5 (5;6) | 5 (4;6) | Ns |
| **Muscle mass and function** | | | |
| **Muscle mass (cm^2^/m^2^)** | 40.83 (39.77;44.18) | 59.11 (55.90;60.86) | p<0.0001 |
| **Handgrip strength (kg)** | 29.15 (27.00;31.00) | 30.33 (26.33;33.60) | Ns |
| **Mid-arm muscle circumference (cm)** | 23.6 (23.2;25.3) | 27.9 (26.4;28.3) | p<0.0001 |
| **Triceps Skinfold Thickness (mm)** | 12.15 (11.00;13.60) | 12.10 (10.20;14.40) | Ns |
| **Gait speed (m/sec)** | 0.92 (0.84;1.00) | 1.02 (0.91;1.07) | Ns |
| **Chair rise (sec)** | 17.95 (16.41;19.60) | 14.68 (14.02;19.28) | Ns |
| **Liver functions** | | | |
| **MELD score** | 11 (10;14) | 11 (9;12) | Ns |
| **Bilirubin (mg/dL)** | 0.70 (0.55;0.96) | 0.38 (0.30;0.57) | Ns |
| **Albumin (g/dL)** | 3.5 (3.4;3.7) | 3.6 (3.4;3.9) | Ns |
| **Total protein (g/dL)** | 7.2 (7.7;7.5) | 7.2 (6.9;7.4) | Ns |
| **Prothrombin time (INR)** | 1.23 (1.16;1.32) | 1.21 (1.15;1.28) | Ns |
| **AST (U/L)** | 46 (43;52) | 52 (47;89) | Ns |
| **ALT (U/L)** | 27 (24;33) | 40 (30;81) | NS |
| **GGT(U/L)** | 131 (96;156) | 113 (92;164) | Ns |
| **Other routine laboratory parameters** | | | |
| **CRP (mg/dL)** | 5.5 (3.4;9.5) | 6.1 (2.8;7.8) | Ns |
| **Hematocrit (%)** | 33.8 (31.4;35.7) | 39.1 (34.2;41.8) | p=0.039 |
| **Creatinine (mg/dL)** | 0,89 (0.81;99) | 0.91 (0.84;1.02) | Ns |
| **Urea (mg/dl)** | 40.0 (35.0;47.0) | 32.0 (30.0;44.0) | Ns |
| **Gut permeability, bacterial translocation, and inflammation** | | | |
| **Zonulin (ng/mL)** | 98.1 (80.8;110.3) | 113.4 (92.4;162.0) | Ns |
| **DAO (U/ml)** | 19.50 (16.23;25.40) | 17.37 (14.09;20.77) | Ns |
| **Calprotectin (μg/g)** | 101.1 (75.4;126.7) | 58.4 (40.8;116.0) | Ns |
| **LBP (µgml)** | 19.21 (17.71;22.30) | 17.73 (16.74;22.02) | Ns |
| **sCD14 (µgml)** | 1.75 (1.67;1.87) | 1.76 (1.70;1.84) | Ns |
| **Muscle biomarkers** | | | |
| **Myostatin (ng/ml)** | 36.45 (34.01;40.47) | 43.27 (36.08;47.36) | Ns |
| **Irisin (µg/ml)** | 1.48 (1.33;1.88) | 1.81 (1.41;2.13) | Ns |
| **FGF21 (ng/ml)** | 0.36 (0.25;0.48) | 0.27 (0.15;0.37) | Ns |
| **IGF-1 (ng/ml)** | 55.89 (44.37;65.70) | 51.74 (43.36;76.35) | Ns |

*^4Note^*^: The data shown are absolute numbers and percentages, or median and 95% confidence interval (lower; upper). P<0.05, values were obtained from the student’s t-test or Mann-Whitney U test.^

^Abbreviations: MELD, model of end-stage liver disease; INR, international normalized ratios; AST, aspartate aminotransferase; ALT, alanine aminotransferase; GGT, gamma-glutamyl-transferase; CRP, C-reactive protein; DAO, diamino-oxidase; LBP, lipopolysaccharide; sCD14, soluble CD14; FGF-21, fibroblast growth factor 21; IGF-1, insulin-like growth factor 1.^

**Table** S 5. Characteristics of female cirrhotic patients with and without sarcopenia. Median (95% CI)

|  | **Cirrhosis sarcopenia (n=14)** | **Cirrhosis no sarcopenia (n=16)** | **P-values** |
| --- | --- | --- | --- |
|  |  |  |  |
| **Age (years)** | 64 (61;68) | 62 (60;67) | Ns |
| **Body mass index (kg/m^2^)** | 24.5 (20.7;28.7) | 31.4 (24.7;32.7) | p=0.01 |
| **Charlson comorbidity index** | 5 (5;6) | 5 (4;6) | Ns |
| **Muscle mass and function** | | | |
| **Muscle mass (cm^2^/m^2^)** | 33.72 (32.19;35.25) | 43.72 (41.21;47.65) | p<0.0001 |
| **Handgrip strength (kg)** | 29.15 (27.00;31.00) | 30.33 (26.33;33.60) | Ns |
| **Mid-arm muscle circumference (cm)** | 22.04 (20.30;24.65) | 24.71 (22.86;27.63) | P=0.04 |
| **Triceps Skinfold Thickness (mm)** | 12.15 (11.00;13.60) | 12.10 (10.20;14.40) | Ns |
| **Gait speed (m/sec)** | 0.92 (0.84;1.00) | 1.02 (0.91;1.07) | Ns |
| **Chair rise (sec)** | 17.95 (16.41;19.60) | 14.68 (14.02;19.28) | Ns |
| **Liver functions** | | | |
| **MELD score** | 11 (10;14) | 11 (9;12) | Ns |
| **Bilirubin (mg/dL)** | 0.70 (0.55;0.96) | 0.38 (0.30;0.57) | Ns |
| **Albumin (g/dL)** | 3.5 (3.4;3.7) | 3.6 (3.4;3.9) | Ns |
| **Total protein (g/dL)** | 7.2 (7.7;7.5) | 7.2 (6.9;7.4) | Ns |
| **Prothrombin time (INR)** | 1.23 (1.16;1.32) | 1.21 (1.15;1.28) | Ns |
| **AST (U/L)** | 46 (43;52) | 52 (47;89) | Ns |
| **ALT (U/L)** | 30.0 (26.0;40.0) | 39.0 (26.0;63.0) | Ns |
| **GGT(U/L)** | 131 (96;156) | 113 (92;164) | Ns |
| **Other routine laboratory parameters** | | | |
| **CRP (mg/dL)** | 5.5 (3.4;9.5) | 6.1 (2.8;7.8) | Ns |
| **Hematocrit (%)** | 30.2 (27.6;35.6) | 34.6 (32.0;36.5) | Ns |
| **Creatinine (mg/dL)** | 0,89 (0.81;99) | 0.91 (0.84;1.02) | Ns |
| **Urea (mg/dl)** | 40.0 (35.0;47.0) | 32.0 (30.0;44.0) | Ns |
| **Gut permeability, bacterial translocation, and inflammation** | | | |
| **Zonulin (ng/mL)** | 98.1 (80.8;110.3) | 113.4 (92.4;162.0) | Ns |
| **DAO (U/ml)** | 19.50 (16.23;25.40) | 17.37 (14.09;20.77) | Ns |
| **Calprotectin (μg/g)** | 101.1 (75.4;126.7) | 58.4 (40.8;116.0) | Ns |
| **LBP (µgml)** | 19.21 (17.71;22.30) | 17.73 (16.74;22.02) | Ns |
| **sCD14 (µgml)** | 1.75 (1.67;1.87) | 1.76 (1.70;1.84) | Ns |
| **Muscle biomarkers** | | | |
| **Myostatin (ng/ml)** | 36.45 (34.01;40.47) | 43.27 (36.08;47.36) | Ns |
| **Irisin (µg/ml)** | 1.48 (1.33;1.88) | 1.81 (1.41;2.13) | Ns |
| **FGF21 (ng/ml)** | 0.36 (0.25;0.48) | 0.27 (0.15;0.37) | Ns |
| **IGF-1 (ng/ml)** | 55.89 (44.37;65.70) | 51.74 (43.36;76.35) | Ns |

*^5Note^*^: The data shown are absolute numbers and percentages, or median and 95% confidence interval (lower; upper). P<0.05, values were obtained from the student’s t-test or Mann-Whitney U test.^

^Abbreviations: MELD, model of end-stage liver disease; INR, international normalized ratios; AST, aspartate aminotransferase; ALT, alanine aminotransferase; GGT, gamma-glutamyl-transferase; CRP, C-reactive protein; DAO, diamino-oxidase; LBP, lipopolysaccharide; sCD14, soluble CD14; FGF-21, fibroblast growth factor 21; IGF-1, insulin-like growth factor 1.^

**Table** S 6. Bile acid and acetate gene abundance distribution between groups. Median (95% CI).

| **Genes** | **Cirrhosis Sarcopenia (n=78)** | **Cirrhosis no sarcopenia (n=38)** | **Corrected P-values** |
| --- | --- | --- | --- |
| **BSH** | 1.05 (0.38;1.65) | 0.63(0.44;1.14) | Ns |
| **3α-HSDH** | 0.35(0.23;0.96) | 0.40(0.18;1.04) | Ns |
| **3β-HSDH** | 0.30(0.22;0.41) | 0.33(0.28;0.43) | Ns |
| **5α-reductase** | 0.21(0.12;0.28) | 0.24(0.15;0.41) | Ns |
| **7α-HSDH** | 0.67(0.50;0.87) | 0.83(0.41;1.04) | Ns |
| **7β-HSDH** | 0.30(0.22;0.46) | 0.39(0.20;0.57) | Ns |
| **12α-HSDH** | 0.11(0.06;0.21) | 0.50(0.11;1.22) | Ns |
| **BaiCD** | 0.98(0.65;1.35) | 0.91(0.65;1.37) | Ns |
| **BaiE** | 0.20(0.12;0.28) | 0.24(0.15;0.41) | Ns |

*^8Note^*^: The data shown are absolute numbers and percentages, or median and 95% confidence interval (lower; upper). P<0.05, values were obtained from the student’s t-test or Mann-Whitney U test. and corrected using multiple testing corrections by the Benjamini-Hochberg method.^

^Abbreviations: BSH, bile salt hydrolase; BaiCD E bile acid inducer CD, and E; 3α-HSDH, 3 alpha-hydroxysteroid dehydrogenases; 3β-HSDH, 3 beta-hydroxysteroid dehydrogenases; 7α-HSDH, 7 alpha-hydroxysteroid dehydrogenases; 7β-HSDH, 7 beta-hydroxysteroid dehydrogenases; 12α-HSDH, 12 alpha-hydroxysteroid dehydrogenases^

**Table** S 7 Serum, stool, and urine metabolite concentrations between groups adjusted for multiple testing . Median (95% CI).

| **Metabolite** | **Cirrhosis sarcopenia (n=78)** | **Cirrhosis no sarcopenia (n=38)** | **Corrected P-values** |
| --- | --- | --- | --- |
| **Serum** | | | |
| **Valine (μmol/l)** | 59.7 (57.0;65.6) | 72.2 (56.0;79.3) | p= 0.01 |
| **Lactate (μmol/l)** | 680.3 (639.7;707.3) | 687.5 (592.6;775.5) | Ns |
| **Alanine (μmol/l)** | 99.2 (92.1;107.4) | 108.7 (99.3;123.6) | Ns |
| **Acetate (μmol/l)** | 16.7 (16.4;17.4) | 17.9 (17.0;19.8) | p=0.03 |
| **Acetone (μmol/l)** | 31.6 (30.7;32.8) | 31.6 (31.1;33.0) | Ns |
| **Ethanol (μmol/l)** | 427.9 (415.9;447.2) | 427.1 (407.1;444.9) | Ns |
| **Succinate (μmol/l)** | 9.1 (8.5;9.3) | 9.1 (8.6;10.1) | Ns |
| **3-HB (μmol/l)** | 11.4 (7.8;17.6) | 11.8 (8.9;19.0) | Ns |
| **Phenylalanine (μmol/l)** | 73.4 (70.4; 75.8) | 77.5 (73.4;82.0) | Ns |
| **Glucose (μmol/l)** | 187.9 (167.0;202.0) | 185.2 (167.4;220.3) | Ns |
| **Hippurate (μmol/l)** | 1.4 (1.3; 1.6) | 1.5 (1.3;2.0) | Ns |
| **Stool** | | | |
| **Valine (μmol/l)** | 68.5 (54.3;80.6) | 68.3 (56.7;77.4) | Ns |
| **Lactate (μmol/l)** | 68.1 (65.5;76.4) | 64.8 (60.8;70.2) | Ns |
| **Alanine (μmol/l)** | 109.8 (93.9;130.3) | 113.9 (96.2;134.4) | Ns |
| **Glycine (μmol/l)** | 34.3 (31.5;44.8) | 32.1 (30.0;39.5) | Ns |
| **Acetate (μmol/l)** | 818.4 (703.5;865.2) | 764.0 (555.6;896.3) | Ns |
| **Succinate (μmol/l)** | 31.0 (25.78;43.1) | 29.9 (27.9;43.6) | Ns |
| **Phenylalanine (μmol/l)** | 78.7 (69.3;95.8) | 80.8 (71.8;90.9) | Ns |
| **Glucose (μmol/l)** | 10.9 (8.5;13.1) | 10.5 (8.5;14.9) | Ns |
| **Acetone (μmol/l)** | 18.9 (17.7;21.8) | 20.8 (17.5;23.3) | Ns |
| **Tyrosine (μmol/l)** | 15.1 (13.3;17.4) | 17.4 (14.2;20.2) | Ns |
| **Isoleucine (μmol/l)** | 47.1 (41.0;56.4) | 50.0 (44.3;57.6) | Ns |
| **Leucine (μmol/l)** | 165.4 (148.9;191.8) | 177.6 (149.8;210.6) | Ns |
| **Urine** | | | |
| **Lactate (μmol/l)** | 37.9 (33.7;43.4) | 34.2 (30.8;38.4) | Ns |
| **Alanine (μmol/l)** | 25.8 (24.1;27.8) | 26.2 (22.2;30.9) | Ns |
| **Acetate (μmol/l)** | 22.9 (21.5;27.4) | 26.0 (22.9;28.0) | Ns |
| **Acetoacetate (μmol/l)** | 15.8 (14.0;19.0) | 16.4 (14.8;18.5) | Ns |
| **Acetone (μmol/l)** | 8.1 (7.62;8.5) | 8.6 (8.2;11.9) | p=0.02 |
| **Succinate (μmol/l)** | 26.2 (24.7;28.0) | 25.6 (23.5;28.4) | Ns |
| **Hippurate (μmol/l)** | 56.4 (43.9;88.4) | 59.3 (40.2;78.3) | Ns |
| **Phenylalanine (μmol/l)** | 55.6 (49.0;70.1) | 70.0 (55.9;90.0) | Ns |
| **Glucose (μmol/l)** | 14.4 (13.7;17.0) | 15.0 (13.3;20.2 | Ns |

*^9Note^*^: The data shown are absolute numbers and percentages, or median and 95% confidence interval (lower; upper). P<0.05 values were obtained from the student’s t-test or Mann-Whitney U test and corrected using multiple testing corrections by the Benjamini-Hochberg method.^

**Table** S 8. Models for predicting sarcopenia in cirrhosis adjusted for MELD and drug use. All values were reported as coefficients and odds ratios (95% CI)

| LASSO predictors | Coefficients | Odd ratios (95% CI) | Adjusted P-values |
| --- | --- | --- | --- |
| Model 1 | | | |
| MAMC | -0.03 | 1.00 (1.01;1.00) | p<0.001 |
| MELD | -0.01 | 1.00 (1.00;0.99) | Ns |
| PPI | -0.15 | 0.87 (0.35;2.08) | Ns |
| β-blocker | 0.10 | 1.10 (0.43;2.75) | Ns |
| Diuretics | 0.59 | 1.80 (0.72;0.45) | Ns |
| Model 2 | | | |
| BMI | -0.21 | 1.04 (1.07;1.02) | p<0.001 |
| MELD | -0.01 | 1.00 (1.01;1.00) | Ns |
| PPI | -0.003 | 0.99 (0.41;2.45) | Ns |
| β-blocker | 0.36 | 1.43 (0.57;3.60) | Ns |
| Diuretics | 0.90 | 2.47 (0.99;6.41) | Ns |
| Model 3 | | | |
| CA: CDCA | -0.25 | 1.64 (1.74;1.01) | p=0.03 |
| MELD | 0.01 | 1.00 (1.01;0.99) | Ns |
| PPI | 0.36 | 1.44 (0.58;3.67) | Ns |
| β-blocker | 0.20 | 1.22 (0.48;3.13) | Ns |
| Diuretics | 0.76 | 2.14 (0.84;5.60) | Ns |
| Model 4 | | | |
| 12-α OH Bas : non 12-α-OH BAs | 1.00 | 2.71 (1.15;7.82) | p=0.04 |
| MELD | 0.02 | 1.00 (0.99;1.02) | Ns |
| PPI | 0.07 | 1.07 (0.46;2.50) | Ns |
| β-blocker | 0.20 | 1.23 (0.51;2.91) | Ns |
| Diuretics | 0.70 | 2.01 (0.86;4.78) | Ns |
| Model 5 | | | |
| T-UDCA: total-sec BAs | -1.75 | 21.38 (264.28;1.94) | p=0.01 |
| MELD | 0.02 | 1.00 (0.99;1.02) | Ns |
| PPI | 0.11 | 1.12 (0.48;2.63) | Ns |
| β-blocker | 0.42 | 1.51 (0.62;3.74) | Ns |
| Diuretics | 0.52 | 1.68 (0.70;4.06) | Ns |
| Model 6 | | | |
| Valine | -0.03 | 1.00 (1.02;1.00) | p=0.04 |
| MELD | 0.05 | 1.00 (0.99;1.00) | Ns |
| PPI | -0.18 | 0.84 (0.33;2.09) | Ns |
| β-blocker | 0.40 | 1.49 (0.57;3.83) | Ns |
| Diuretics | 0.44 | 1.56 (0.57;4.34) | Ns |

*^10Note^*^. p-values obtained through multivariate logistic regression and adjusted for the severity of liver disease and drug use.^

^Abbreviations: MAMC, mid-arm muscle circumference; MELD, model of end-stage liver disease; BMI, body mass index; PPI, proton pump inhibitor; CA: CDCA, the ratio of cholic acid to chenodeoxycholic acid; 12-α OH BAs: non-12-α-OH Bas, 12 alpha-hydroxylated to non-12 alpha-hydroxylated BAs; T-UDCA: total-sec Bas, total ursodeoxycholic acid to total secondary BAs.^


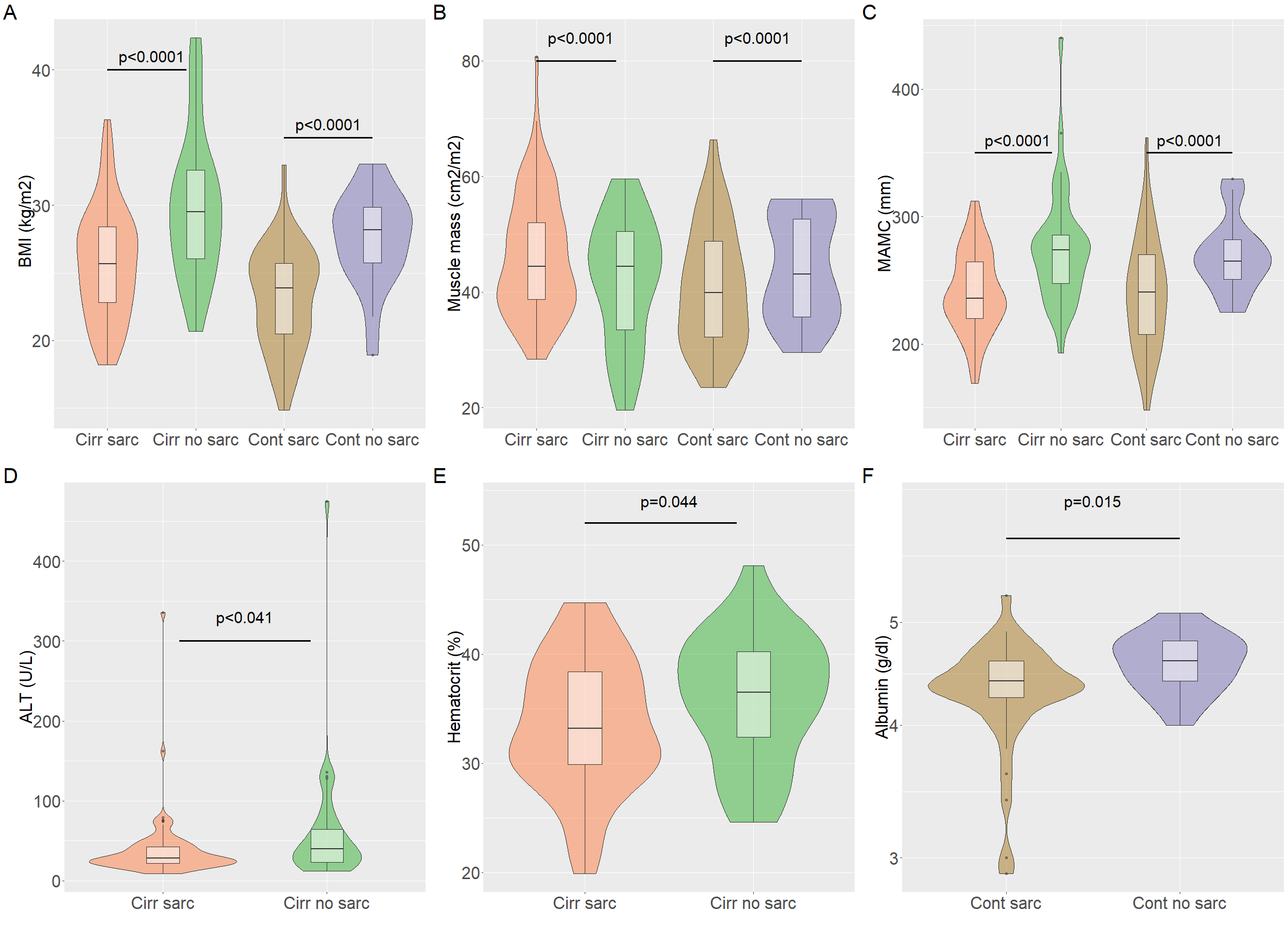


**Fig** S 1. Altered anthropometric measurements (A) and altered muscle mass biomarkers (B-C) between cirrhotic patients with and without sarcopenia and controls with and without sarcopenia. Altered laboratory markers between cirrhotic patients with and without sarcopenia (D-F).


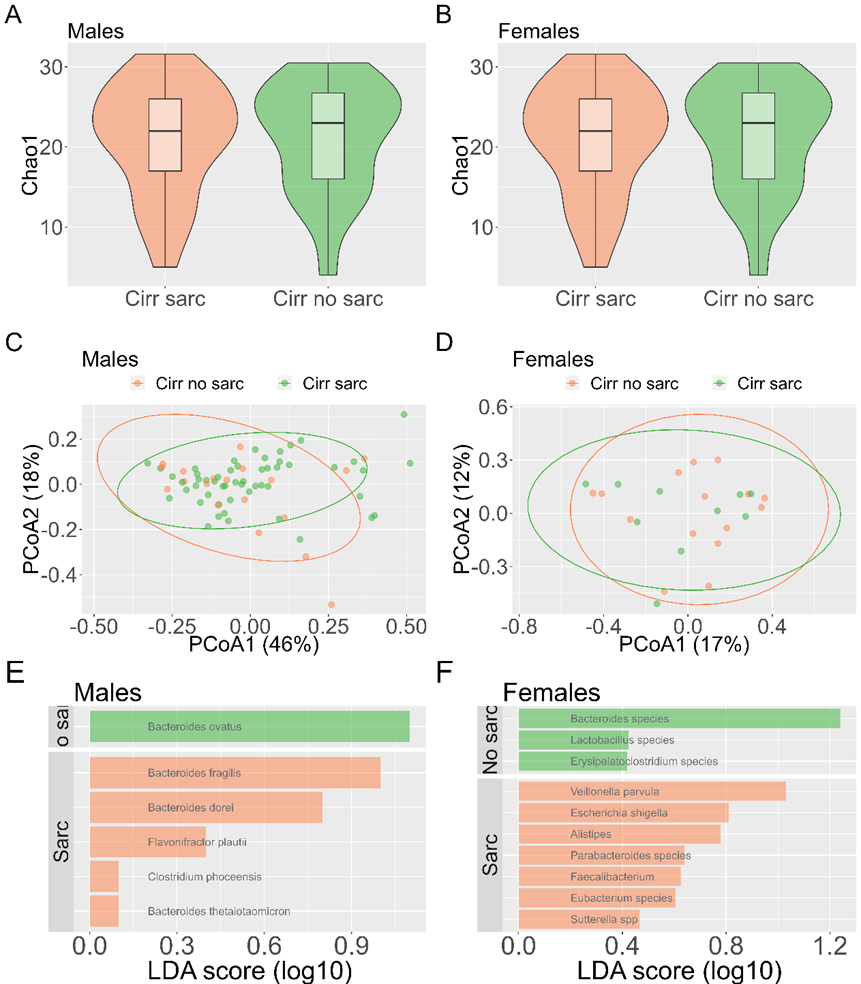


**Fig S 2** Chao1 index as a measure of alpha diversity between male cirrhotic patients with and without sarcopenia (A). Chao1 index as a measure of alpha diversity between female cirrhotic patients with and without sarcopenia (B). Bray-Curtis as a measure of beta-diversity between male cirrhotic patients with and without sarcopenia (C). Bray-Curtis as a measure of beta-diversity between male cirrhotic patients with and without sarcopenia (D). Bacteria identified by regularized logistic least absolute shrinkage and selection operator (LEfSe) to be associated with male cirrhotic patients with and without sarcopenia (E). Bacteria identified by regularized logistic least absolute shrinkage and selection operator (LEfSe) to be associated with male cirrhotic patients with and without sarcopenia (F).


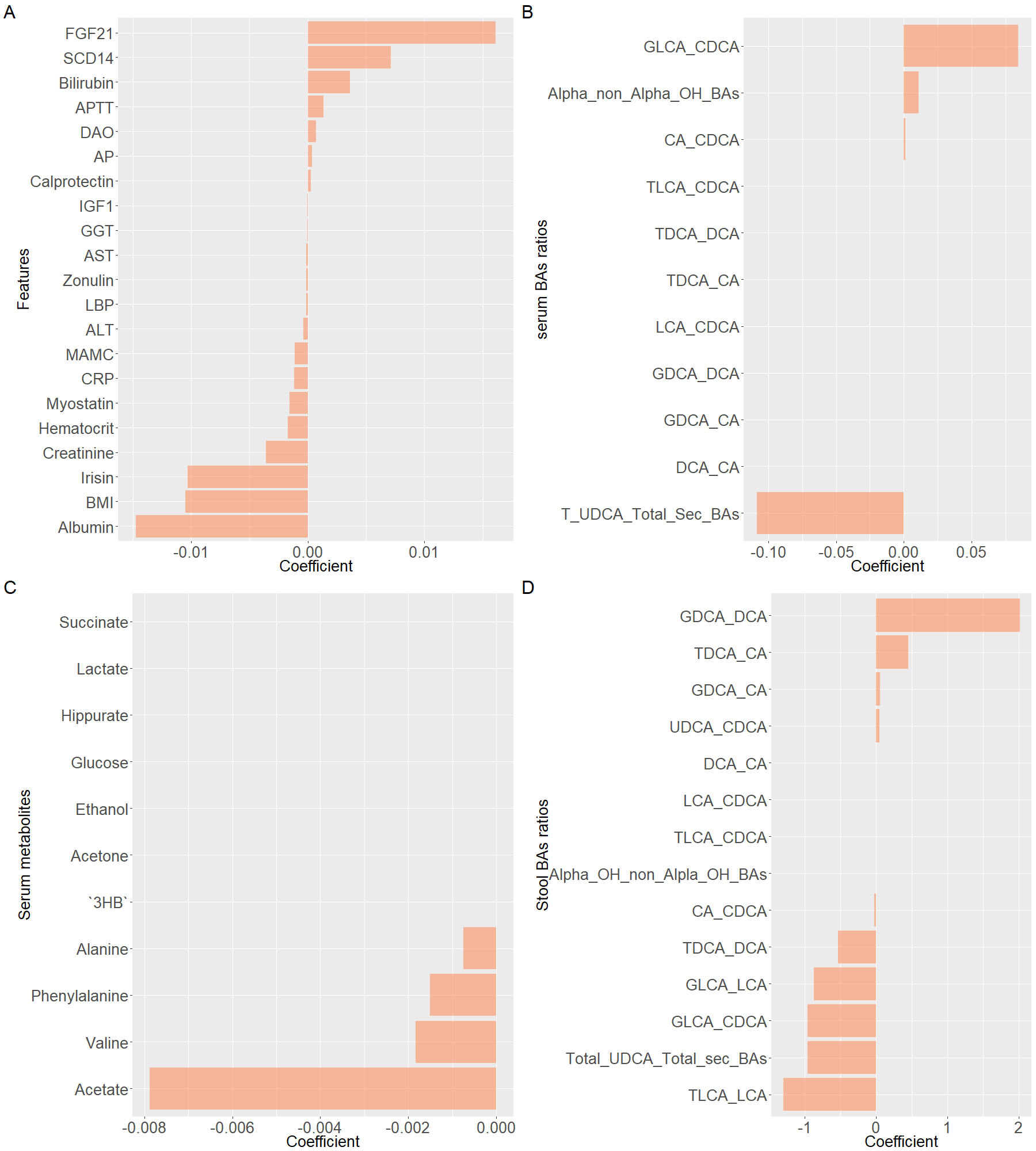


**Fig** S 3 Features identified by LASSO regression as predictors of sarcopenia in liver cirrhosis.


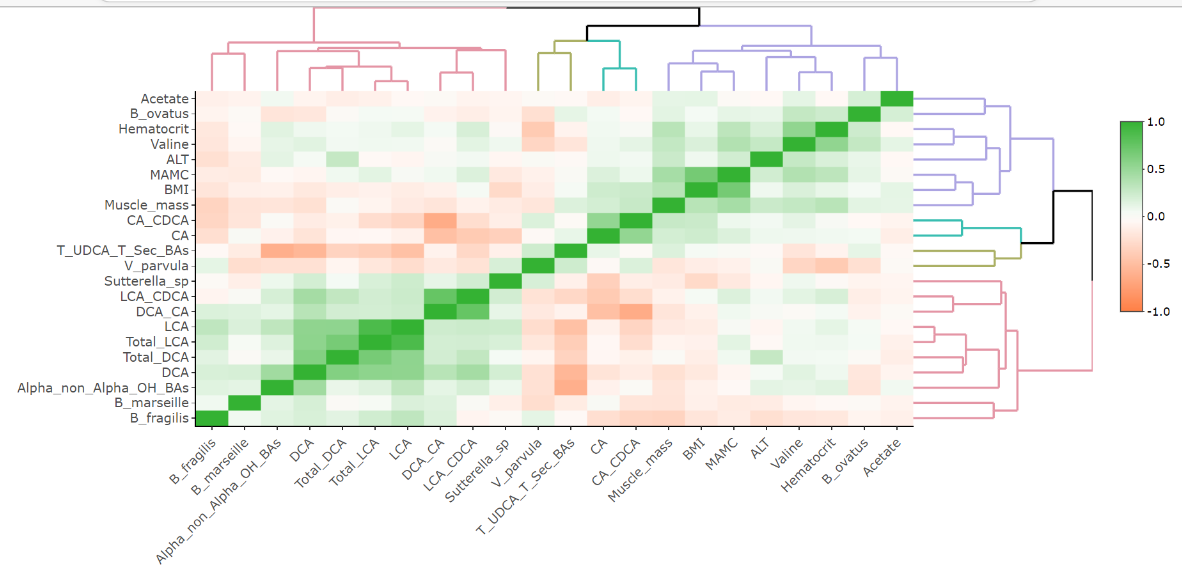


**Fig** S 4 Correlation between clinical parameters, bacterial OTUs, BAs, and metabolites. Green and Indian red indicate positive and negative correlations, respectively.

# References

1. Stadlbauer V, Horvath A, Ribitsch W, Schmerböck B, Schilcher G, Lemesch S, et al. Structural and functional differences in gut microbiome composition in patients undergoing haemodialysis or peritoneal dialysis. Sci Rep. 2017;7(1):15601.

2. Caporaso JG, Kuczynski J, Stombaugh J, Bittinger K, Bushman FD, Costello EK, et al. QIIME allows analysis of high-throughput community sequencing data. Nat Methods. 2010;7(5):335-6.

3. Callahan BJ, McMurdie PJ, Rosen MJ, Han AW, Johnson AJ, Holmes SP. DADA2: High-resolution sample inference from Illumina amplicon data. Nat Methods. 2016;13(7):581-3.

4. Quast C, Pruesse E, Yilmaz P, Gerken J, Schweer T, Yarza P, et al. The SILVA ribosomal RNA gene database project: improved data processing and web-based tools. Nucleic Acids Res. 2013;41(Database issue):D590-6.

5. Database resources of the National Center for Biotechnology Information. Nucleic Acids Res. 2016;44(D1):D7-19.

6. Zakrzewski M, Proietti C, Ellis JJ, Hasan S, Brion MJ, Berger B, et al. Calypso: a user-friendly web-server for mining and visualizing microbiome-environment interactions. Bioinformatics. 2017;33(5):782-3.

7. Li K, Buchinger TJ, Bussy U, Fissette SD, Johnson NS, Li W. Quantification of 15 bile acids in lake charr feces by ultra-high performance liquid chromatography-tandem mass spectrometry. J Chromatogr B Analyt Technol Biomed Life Sci. 2015;1001:27-34.

8. Kakiyama G, Muto A, Takei H, Nittono H, Murai T, Kurosawa T, et al. A simple and accurate HPLC method for fecal bile acid profile in healthy and cirrhotic subjects: validation by GC-MS and LC-MS. J Lipid Res. 2014;55(5):978-90.

9. Reisinger AC, Posch F, Hackl G, Marsche G, Sourij H, Bourgeois B, et al. Branched-Chain Amino Acids Can Predict Mortality in ICU Sepsis Patients. Nutrients. 2021;13(9).

10. Alkan HF, Walter KE, Luengo A, Madreiter-Sokolowski CT, Stryeck S, Lau AN, et al. Cytosolic Aspartate Availability Determines Cell Survival When Glutamine Is Limiting. Cell Metab. 2018;28(5):706-20.e6.

11. Ye J, Coulouris G, Zaretskaya I, Cutcutache I, Rozen S, Madden TL. Primer-BLAST: a tool to design target-specific primers for polymerase chain reaction. BMC Bioinformatics. 2012;13:134.

12. Mullish BH, Pechlivanis A, Barker GF, Thursz MR, Marchesi JR, McDonald JAK. Functional microbiomics: Evaluation of gut microbiota-bile acid metabolism interactions in health and disease. Methods. 2018;149:49-58.
